# Cytotoxic Alkaloids from the Stem of *Xylopia laevigata*

**DOI:** 10.3390/molecules21070890

**Published:** 2016-07-08

**Authors:** Leociley R. A. Menezes, Cinara O. D´Sousa Costa, Ana Carolina B. da C. Rodrigues, Felipe R. do E. Santo, Angelita Nepel, Lívia M. Dutra, Felipe M. A. Silva, Milena B. P. Soares, Andersson Barison, Emmanoel V. Costa, Daniel P. Bezerra

**Affiliations:** 1 Department of Chemistry, Federal University of Sergipe, São Cristóvão 49100-000, Sergipe, Brazil; leociley@gmail.com; 2 Gonçalo Moniz Institute, Oswaldo Cruz Foundation (IGM-FIOCRUZ), Salvador 40000-000, Bahia, Brazil; cnbiologa@gmail.com (C.O.D.C.); anacarolinabcr@gmail.com (A.C.B.d.C.R.); felipe.rosario@live.com (F.R.d.E.S.); milenabpsoares@gmail.com (M.B.P.S.); 3 NMR Center, Federal University of Paraná, Curitiba 80000-000, Paraná, Brazil; angelneppel@gmail.com (A.N.); liviamacedodutra@yahoo.com.br (L.M.D.); anderbarison@gmail.com (A.B.); 4 Department of Chemistry, Federal University of Amazonas, Manaus 69000-000, Amazonas, Brazil; felipesaquarema@bol.com.br; 5 Center of Biotechnology and Cell therapy, Hospital São Rafael, Salvador 40000-000, Bahia, Brazil

**Keywords:** *Xylopia laevigata*, Annonaceae, alkaloids, cytotoxicity, apoptosis

## Abstract

*Xylopia laevigata* (Annonaceae), known locally as “meiú” or “pindaíba”, is widely used in folk medicine in Northeastern Brazil. In the present work, we performed phytochemical analyses of the stem of *X. laevigata*, which led to the isolation of 19 alkaloids: (−)-roemerine, (+)-anonaine, lanuginosine, (+)-glaucine, (+)-xylopine, oxoglaucine, (+)-norglaucine, asimilobine, (−)-xylopinine, (+)-norpurpureine, (+)-*N*-methyllaurotetanine, (+)-norpredicentrine, (+)-discretine, (+)-calycinine, (+)-laurotetanine, (+)-reticuline, (−)-corytenchine, (+)-discretamine and (+)-flavinantine. The in vitro cytotoxic activity toward the tumor cell lines B16-F10 (mouse melanoma), HepG2 (human hepatocellular carcinoma), K562 (human chronic myelocytic leukemia) and HL-60 (human promyelocytic leukemia) and non-tumor peripheral blood mononuclear cells (PBMCs) was tested using the Alamar Blue assay. Lanuginosine, (+)-xylopine and (+)-norglaucine had the highest cytotoxic activity. Additionally, the pro-apoptotic effects of lanuginosine and (+)-xylopine were investigated in HepG2 cells using light and fluorescence microscopies and flow cytometry-based assays. Cell morphology consistent with apoptosis and a marked phosphatidylserine externalization were observed in lanuginosine- and (+)-xylopine-treated cells, suggesting induction of apoptotic cell death. In addition, (+)-xylopine treatment caused G_2_/M cell cycle arrest in HepG2 cells. These data suggest that *X. laevigata* is a potential source for cytotoxic alkaloids.

## 1. Introduction

*Xylopia laevigata* (Mart.) R.E. Fries, a typical plant of the family Annonaceae, is popularly known as “meiú” or “pindaíba”. Its leaves and flowers are used in folk medicine in Northeastern Brazil to treat painful disorders, heart diseases and inflammatory conditions [1].

Chemical studies have previously been reported for this species. Chemical analysis of the stems of *X. laevigata* resulted in the isolation of the *ent*-kaurane diterpenoids *ent*-kaur-16-en-19-oic acid, 4-*epi*-kaurenic acid, *ent*-16β-hydroxy-17-acetoxy-kauran-19-al, *ent*-3β-hydroxykaur-16-en-19-oic acid and *ent*-16β,17-dihydroxy-kauran-19-oic acid, as well as spathulenol and a mixture of β-sitosterol, stigmasterol and campesterol [2]. The chemical composition of essential oil from the leaves of *X. laevigata* included γ-muurolene, δ-cadinene, germacrene B, α-copaene, germacrene D, bicyclogermacrene and (*E*)-caryophyllene as the major constituents [1,3,4,5]. The essential oil from its fresh fruits contained limonene, α-pinene and β-pinene as the major compounds [6]. Moreover, isoquinoline alkaloids were also found in its leaves [7]. The terpenoids and essential oil exhibited trypanocidal, larvicidal, antifungal, antibacterial, antioxidant, antitumor, anti-inflammatory and antinociceptive activities [1,2,3,4,5]. In this report, 19 alkaloids were isolated from the stem of *X. laevigata*, and their in vitro cytotoxic activities towards tumor cell lines and non-tumor cells were investigated.

## 2. Results and Discussion

**Figure 1:**
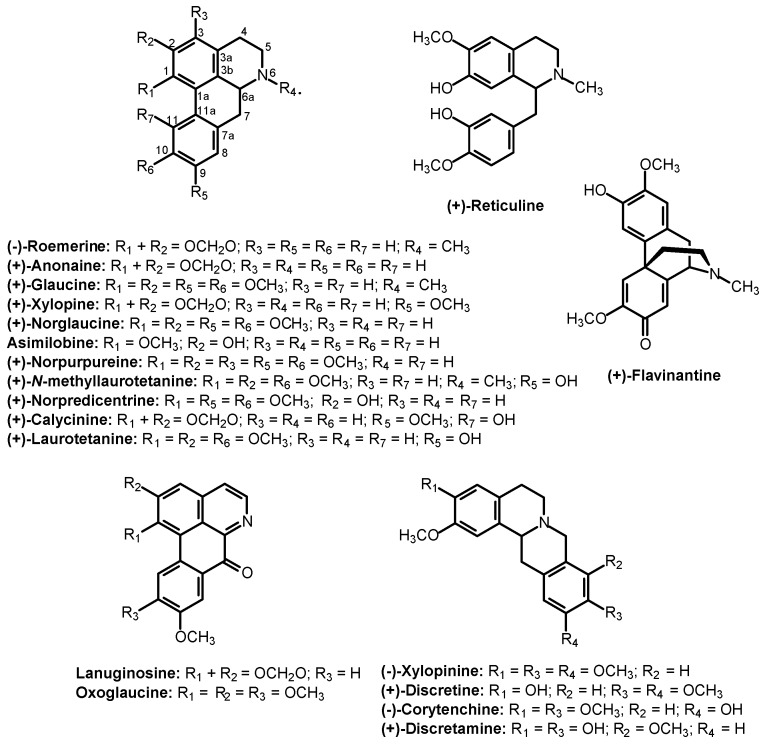
Chemical structures of the alkaloids from the stem of *Xylopia laevigata*.

Phytochemical analysis of the stem of *X. laevigata* resulted in the isolation of 19 alkaloids, including 11 aporphines, namely, (−)-oemerine [8], (+)-anonaine [9], (+)-glaucine [10], (+)-xylopine [11], (+)-norglaucine [12], asimilobine [9], (+)-norpurpureine [13], (+)-*N*-methyllaurotetanine [14], (+)-norpredicentrine [15], (+)-calycinine [16] and (+)-laurotetanine [7]; two oxoaporphines, namely, lanuginosine [17] and oxoglaucine [18]; four tetrahydroprotoberberinic alkaloids, namely, (−)-xylopinine [11], (+)-discretine [7], (−)-corytenchine [11] and (+)-discretamine [9]; one benziltetrahydroisoquinoline, namely, (+)-reticuline [19]; and one morphinandienone, namely, (+)-flavinantine [20]. The chemical structures are presented in Figure 1. All compounds were identified by a series of spectrometric data, including MS, NMR 1D and 2D (COSY, HSQC, HMBC and NOE), as well as by comparison with data reported in the literature. The alkaloids (−)-roemerine, (+)-anonaine, (+)-glaucine, (+)-xylopine, (+)-norglaucine, asimilobine, (+)-norpurpureine, (+)-*N*-methyllaurotetanine, (+)-norpredicentrine, (+)-calycinine, oxoglaucine, (−)-xylopinine, (−)-corytenchine, (+)-discretamine and (+)-flavinantine are described in *X. laevigata* for the first time in this report. The complete and unequivocal ^1^H- and ^13^C-NMR data for isolated compounds are described in the Supplementary Materials (Tables S1 and S2) as well as MS, ^1^H- and ^13^C-NMR spectra (Figures S1–S46). In Tables S1 and S2, only ^1^H- and ^13^C-NMR data were listed while some of the other isolated compounds were recently described in the literature, such as anonaine, asimilobine and discretamine [9].

The cytotoxicity of the alkaloids towards the tumor cell lines B16-F10 (mouse melanoma), HepG2 (human hepatocellular carcinoma), K562 (human chronic myelocytic leukemia) and HL-60 (human promyelocytic leukemia) and non-tumor peripheral blood mononuclear cells (PBMCs) was assessed after 72 h of incubation using the Alamar Blue assay (Table 1). Asimilobine was not tested due to the small amount of sample available. Lanuginosine, (+)-xylopine and (+)-norglaucine were the most active compounds, presenting 50% inhibitory concentration (IC_50_) values below 4 μg/mL in at least one of the tumor cell lines tested, while (+)-anonaine, oxoglaucine, (+)-norpurpureine, (+)-discretine, (+)-calycinine, (+)-reticuline and (+)-discretamine showed IC_50_ values below 20 μg/mL in at least one tested tumor cell line. On the other hand, (−)-roemerine, (+)-glaucine, (−)-xylopinine, (+)-*N*-methyllaurotetanine, (+)-norpredicentrine, (+)-laurotetanine, (−)-corytenchine and (+)-flavinantine showed no cytotoxic activity at the concentrations tested (IC_50_ > 25 μg/mL). Doxorubicin, used as the positive control, presented IC_50_ values of 0.08 and 0.15 μg/mL for HepG2 and K562 tumor cell lines, respectively. In our preclinical cytotoxic drug screening program, pure compounds with IC_50_ values below 4 μg/mL in tumor cell line assays are considered promising [1,21]. Therefore, the alkaloids lanuginosine, (+)-xylopine and (+)-norglaucine presented promising results. The selectivity indexes (SI) were 3.1, 2.2 and 1.0 for lanuginosine, (+)-xylopine and (+)-norglaucine, respectively (the SI was calculated using the following formula: SI = IC_50_[PBMC]/IC_50_[HL-60]). Doxorubicin had a SI of 27.4.

Therefore, a new series of experiments was conducted to investigate the pro-apoptotic action of lanuginosine and (+)-xylopine. For this, HepG2 cells were incubated for 24 h with the drugs at concentrations of 10 and 20 μg/mL for lanuginosine and 5 and 10 μg/mL for (+)-xylopine. This period of incubation was chosen to allow the cells to pass through all phases of the cell cycle. This cell line was used due to its sensitivity to lanuginosine and (+)-xylopine cytotoxicity, and these concentrations were chosen based on the IC_50_ values determined in this cell line (3.89 μg/mL for lanuginosine and 1.87 μg/mL for (+)-xylopine). Although (+)-norglaucine had also shown cytotoxic effects, its ability to induce apoptosis was not investigated here because it had been previously studied for its pro-apoptotic effect, and no effect was found [22]. Herein, the pro-apoptotic effect of lanuginosine and (+)-xylopine was assessed for the first time.

**Figure 2:**
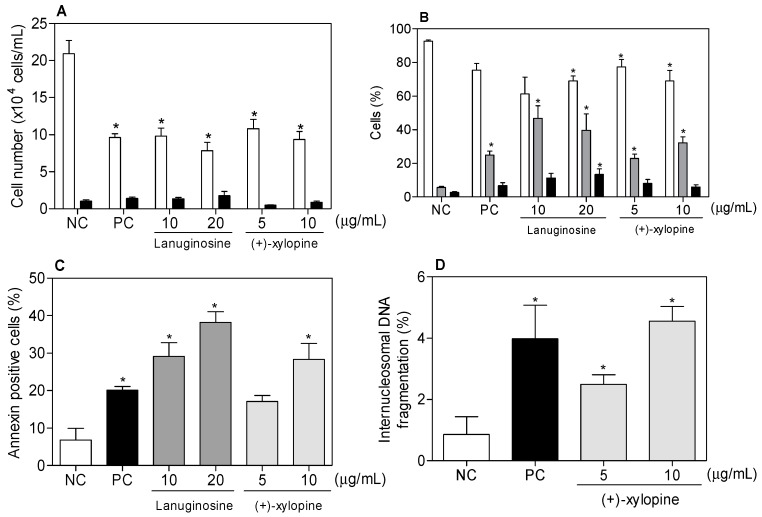
Effect of lanuginosine and (+)-xylopine on the viability of human hepatocellular carcinoma (HepG2) cells after 24 h of incubation.(**A**): Cell viability measured by trypan blue exclusion assay–viable cells (white bars) and non-viable cells (black bars); (**B**): Cell viability measured by fluorescence microscopy using acridine orange/ethidium bromide staining–viable cells (white bars), apoptotic cells (grey bars) and necrotic cells (black bars); (**C**): Cell viability measured by flow cytometry using FITC annexin V and (**D**) Internucleosomal DNA fragmentation measured by flow cytometry using propidium iodide and triton X-100. Negative control (**NC**) was treated with the vehicle (0.2% DMSO) used for diluting the tested substance. Doxorubicin (1 μg/mL) was used as the positive control (**PC**). The data are presented as the mean values ± S.E.M. from at least three independent experiments performed in duplicate. For flow cytometric analysis, ten thousand events were evaluated per experiment and cellular debris was omitted from the analysis. * *p* < 0.05 compared to negative control by ANOVA followed by the Student-Newman-Keuls test.

**Figure 3:**
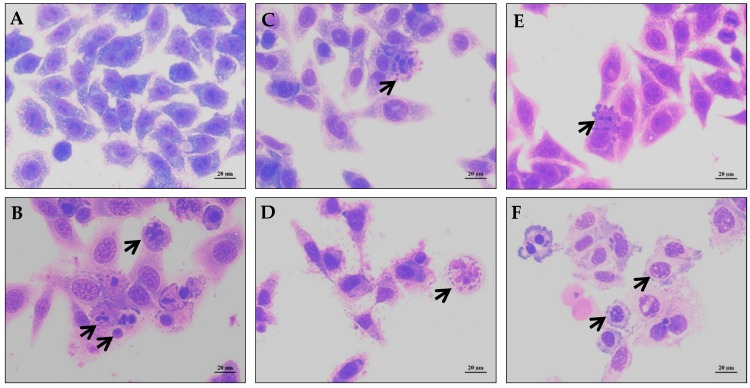
Effect of lanuginosine and (+)-xylopine on the cell morphology of human hepatocellular carcinoma (HepG2) cells. The cells were stained with May-Grünwald-Giemsa stain and analyzed by optical microscopy after 24 h of incubation. Negative control (**A**) was treated with the vehicle (0.2% DMSO) used for diluting the tested substance; Doxorubicin (1 μg/mL) was used as the positive control (**B**); Lanuginosine was used at concentrations of 10 (**C**); and 20 μg/mL (**D**); (+)-xylopine at concentrations of 5 (**E**) and 10 μg/mL (**F**). Black arrows show chromatin condensation or DNA fragmentation.

When tested using a Trypan Blue dye exclusion evaluation method, both alkaloids were able to reduce the number of viable cells without increasing the number of non-viable cells (*p* < 0.05, Figure 2A). In the Trypan Blue assay, the non-viable cells represent the cells without membrane integrity, suggesting that the compounds did not affect the cell membrane integrity. In Acridine Orange and ethidium bromide (AO/EB) staining, an increased number of apoptotic cells were observed (*p* < 0.05, Figure 2B). Only lanuginosine, at the higher concentration, significantly increased the percentage of necrotic cells (*p* < 0.05). Morphological analysis using May-Grünwald-Giemsa staining showed that cells treated with lanuginosine and (+)-xylopine also presented morphology consistent with apoptosis, including chromatin condensation and fragmentation of the nuclei (Figure 3). In addition, phosphatidylserine externalization was measured in lanuginosine- and (+)-xylopine-treated cells after 24 h of incubation (Figure 2C). A significant increase in phosphatidylserine exposure was observed (*p* < 0.05), without affecting the cell membrane integrity (data not shown), which is also compatible with changes associated with apoptotic cell death. Incubation with doxorubicin, used as the positive control, also induced apoptotic characteristics in the cells.

Cell cycle arrest is a common cause of cell growth inhibition. To determine whether (+)-xylopine cytotoxicity involves alterations in cell cycle progression, analysis of cell cycle distribution by flow cytometry was included in the study. Lanuginosine was not tested due to the small amount of sample availability. All subdiploid DNA (sub-G_1_ phase) was considered to be internucleosomal DNA fragmentation. The results of the effect of (+)-xylopine on cell cycle distribution showed that the total number of cells in the G_2_/M phase increased, indicating cell cycle arrest during this phase (Table 2). In addition to the increased number of cells in G_2_/M, an increase in the internucleosomal DNA fragmentation (*p* < 0.05, Figure 2D) was also observed. 

## 3. Experimental Section

### 3.1. Botanical Material

The stem of *X. laevigata* was collected in “Serra de Itabaiana”, located between the cities of Itabaiana and Areia Branca (coordinates: 10°44’50’’S, 37°20’24’’W), Sergipe, Brazil, in February 2013. The identity of the plant was confirmed by Dr. Ana Paula do N. Prata, Department of Biology, Federal University of Sergipe, Brazil, and a voucher specimen (#26805) has been deposited in the Herbarium of the Federal University of Sergipe. The authors have authorization from the Chico Mendes Institute for Biodiversity Conservation from the Brazilian Ministry of the Environment for plant collection (#25637-1). This work was performed according to a special authorization for access to genetic resources in Brazil #010240/2013-6, issued by CNPq/MCTI.

### 3.2. Phytochemical Analyses

#### 3.2.1. General Procedures

Optical rotations were measured in chloroform at room temperature on a P-2000 polarimeter at 589 nm (sodium D line) (JASCO, Hachioji, Japan). One-dimensional (1D) and two-dimensional (2D) nuclear magnetic resonance (NMR) experiments were acquired in CDCl_3_, CDCl_3_ + drops of CD_3_OD or CD_3_OD at 303 K on a AVANCE 400 NMR spectrometer (Bruker, Karlsruh, Germany) operating at 9.4 Tesla, observing the ^1^H and ^13^C nuclei at 400 and 100 MHz, respectively. The spectrometer was equipped with either a 5-mm multinuclear direct detection probe (1D NMR experiments) or a 5-mm multinuclear inverse detection probe (1D nuclear Overhauser effect [NOE] and 2D NMR experiments), both with a *z*-gradient. Low-resolution mass spectra (LR-MS) were obtained on a TSQ Quantum Access ultra-high-performance liquid chromatograph (Thermo, Waltham, MA, USA) coupled with a mass spectrometer using electrospray ionization (ESI) and atmospheric pressure chemical ionization (APCI) sources in the positive ion mode. Silica gel 60 (70–230 mesh) was used for column chromatography (CC), while silica gel 60 F_254_ was used for analytical (0.25 mm) and preparative (1.00 mm) thin layer chromatography (TLC). Compounds were visualized by exposure under ultraviolet (UV_254/365_) light or by spraying with *p*-anisaldehyde followed by heating on a hot plate and by spraying with Dragendorff’s reagent. All chemicals and solvents were of analytical grade.

#### 3.2.2. Extraction and Isolation

The dried and powdered stem of *X. laevigata* (1400.00 g) was extracted with hexane (5 L, 25 °C, five times) followed by methanol (5 L, 25 °C, five times) to yield after solvent removal the corresponding hexane (18.77 g) and methanol (87.79 g) extracts. TLC analysis indicated a high concentration of alkaloids in the methanol extract. Therefore, an aliquot of the methanol extract (84.00 g) was further subjected to an acid-base extraction to give alkaloid (0.93 g) and neutral (5.50 g) fractions. An aliquot of the alkaloid fraction (0.73 g), previously treated with a 10% sodium bicarbonate solution, was subjected to CC using silica gel. Subsequently, it was eluted with increasing concentrations of dichloromethane in *n*-hexane (100:0 to 10:90, *v/v*), followed by ethyl acetate in dichloromethane (100:0 to 10:90, *v/v*) and methanol in ethyl acetate (100:0 to 50:50, *v/v*), affording 218 fractions (30 mL each). These fractions were evaluated and pooled according to TLC analysis, yielding 26 groups (G1-G26).

Group G2 (3.8 mg) from *n*-hexane–dichloromethane (80:20 and 70:30, *v/v*) afforded (−)-roemerine (3.8 mg). Group G3 (117.4 mg) from *n*-hexane-dichloromethane (70:30 and 60:40, *v/v*) was submitted to a new silica gel CC process and were eluted with increasing concentrations of dichloromethane in *n*-hexane (100:0 to 20:80, *v/v*), followed by ethyl acetate in dichloromethane (100:0 to 30:70, *v/v*) and methanol in ethyl acetate (100:0 to 50:50, *v/v*), affording 51 subfractions (30 mL each) that were evaluated and pooled according to TLC analysis and which resulted in 11 groups (G3.1 to G3.11). Group G3.3 (41.3 mg) was purified by preparative TLC, eluted with dichloromethane-methanol (95:05, three times), to yield (+)-anonaine (7.0 mg), lanuginosine (5.0 mg), (+)-glaucine (1.0 mg) and (+)-xylopine (13.4 mg). Group G3.4 (37.5 mg) was also subjected to preparative TLC eluted with dichloromethane-methanol (95:05, three times), affording oxoglaucine (8.3 mg), (+)-norglaucine (8.2 mg) and (−)-xylopinine (8.0 mg). Group G4 (44.0 mg) from *n*-hexane-dichloromethane (60:40 and 50:50, *v/v*) was also subjected to preparative TLC and eluted with dichloromethane-methanol (95:05, twice), resulting in asimilobine (0.7 mg), (+)-norpurpureine (4.3 mg) and (+)-*N*-methyllaurotetanine (5.4 mg). Group G5 (58.2 mg) from *n*-hexane–dichloromethane (50:50, *v/v*) was also subjected to preparative TLC and eluted with dichloromethane-methanol (95:05, twice), resulting in (+)-norpredicentrine (7.1 mg) and (+)-discretine (21.9 mg). Group G7 (44.1 mg) from *n*-hexane-dichloromethane (50:50 and 20:80, *v/v*) was also subjected to preparative TLC and eluted with dichloromethane–methanol (95:05, three times), giving (+)-calycinine (1.5 mg), (+)-laurotetanine (8.2 mg), (+)-reticuline (14.1 mg) and (−)-corytenchine (1.6 mg). Group G9 (25.2 mg) from dichloromethane–ethyl acetate (100:0 and 95:05, *v/v*) was also subjected to a preparative TLC and eluted with dichloromethane–methanol (95:05, three times), resulting in (+)-discretamine (3.5 mg) and (+)-flavinantine (2.4 mg). These compounds were analyzed using HPLC to confirm the purity degree. All compounds presented >90% purity.

### 3.3. Biological Evaluation

#### 3.3.1. Cells

B16-F10, HepG2, K562 and HL-60 tumor cell lines were kindly donated by Hospital A.C. Camargo, São Paulo, Brazil. Cells were maintained in RPMI 1640 medium (Gibco-BRL, Gaithersburg, MD, USA) with 10% fetal bovine serum (Cultilab, Campinas, Brazil), 2 mM L-glutamine (Vetec Química Fina, Duque de Caxias, Brazil) and 50 μg/mL gentamycin (Novafarma, Anápolis, Brazil). Adherent cells were collected by treatment with 0.25% trypsin-EDTA solution (Gibco-BRL). All cell lines were cultured in flasks at 37 °C in 5% CO_2_ and sub-cultured every 3–4 days to maintain exponential growth. All cell lines were tested for mycoplasma using a Mycoplasma Stain Kit (Sigma-Aldrich, St. Louis, MO, USA) to confirm that the cells were free from contamination.

Heparinized blood was collected from 20- to 35-year-old, healthy non-smokers who had not taken any drugs for at least 15 days prior to sampling, and the peripheral blood mononuclear cells (PBMCs) were isolated using a Ficoll density gradient in a Ficoll-Paque Plus (GE Healthcare Bio-Sciences AB, Uppsala, Sweden). PBMCs were washed and resuspended at a concentration of 0.3 × 10^6^ cells/mL in RPMI 1640 medium with 20% fetal bovine serum, 2 mM L-glutamine and 50 μg/mL gentamycin at 37 °C with 5% CO_2_. Concanavalin A (ConA, Sigma-Aldrich Co.) was used as a mitogen to trigger cell division in T-lymphocytes. ConA (10 μg/mL) was added at the beginning of culture, and the cells were treated with the test compounds after 24 h. Cell viability was examined using a trypan blue exclusion assay for all experiments. Over 90% of the cells were viable at the beginning of the culture. The Research Ethics Committee of the Oswaldo Cruz Foundation (Salvador, Bahia, Brazil) approved the experimental protocol (#031019/2013). All participants signed a written informed consent to participate in the study.

#### 3.3.2. In Vitro Cytotoxic Activity Assay

Cell viability was quantified using the Alamar Blue assay, according to Ahmed et al. [23]. Cells were placed in 96-well plates for all experiments (0.7 × 10^5^ cells/mL for adherent cells or 0.3 × 10^6^ cells/mL for suspended cells in 100 μL of medium). After 24 h, the alkaloids (in a range of eight different concentrations varying from 0.19 to 25 μg/mL) were dissolved in dimethyl sulfoxide (DMSO, Sigma-Aldrich Co.), and the solution was added to each well and incubated for 72 h. Doxorubicin (purity ≥ 95.0%, doxorubicin hydrochloride, Laboratory IMA S.A.I.C., Buenos Aires, Argentina) was used as the positive control (0.08–5 μg/mL). Negative controls received the vehicle that was used for diluting the alkaloids (0.5% DMSO). Four (for cell lines) or 24 h (for PBMCs) before the end of incubation, 20 μL of a stock solution (0.312 mg/mL) of the Alamar Blue (resazurin, Sigma-Aldrich Co.) was added to each well. The absorbance at 570 nm and 600 nm was measured using a SpectraMax 190 Microplate Reader (Molecular Devices, Sunnyvale, CA, USA), and the drug effect was quantified as the percentage of control absorbance. 

The following set of experiments was performed to investigate the mechanisms involved in the cytotoxic action of the alkaloids lanuginosine and (+)-xylopine. In all experiments, 2 mL of a HepG2 cell solution (0.7 × 10^5^ cells/mL) was placed in 24-well plates and incubated overnight to allow the cells to adhere to the plate surface. The cells were then treated for 24 h with the alkaloids lanuginosine (10 and 20 μg/mL) and (+)-xylopine (5 and 10 μg/mL). The negative control was treated with the vehicle (0.2% DMSO) used for diluting the alkaloids. Doxorubicin (1 μg/mL) was used as the positive control. Cell viability was assessed for all experiments using a Trypan Blue exclusion assay. 

#### 3.3.3. Morphological Analysis with May-Grünwald-Giemsa Staining

To evaluate alterations in morphology, cells were cultured under a coverslip, fixed with methanol for 30 s and stained with May-Grünwald-Giemsa stain. Morphological changes were examined by light microscopy (Olympus BX41, Tokyo, Japan) using Image-Pro software (Media Cybernetics, Rockville, MD, USA).

#### 3.3.4. Morphological Analysis with Acridine Orange/Ethidium Bromide Staining

Cells were pelleted and resuspended in saline (25 μL). Then, 1 μL of aqueous solution of AO/EB (100 μg/mL, Sigma-Aldrich Co.) was added, and the cell viability changes were observed under a fluorescence microscope (Olympus BX41). In this assay, AO permeates all cells and makes the nuclei appear green. EB is only taken up by cells when the cytoplasmic membrane integrity is lost and stains the nucleus red. EB also dominates over AO. Thus, live cells have a normal green nucleus; early apoptotic cells have a bright green nucleus with condensed or fragmented chromatin; late apoptotic cells display condensed and fragmented orange chromatin; cells that have died from direct necrosis have a structurally normal orange nucleus. Three hundred cells were counted per sample and classified as viable, apoptotic or necrotic.

#### 3.3.5. Annexin Assay

Phosphatidylserine externalization was analyzed by flow cytometry. A FITC annexin V apoptosis detection kit (BD Biosciences, San Jose, CA, USA) was used to determine cell viability (viable, early apoptotic, late apoptotic and necrotic cells). Cells were washed twice with saline and then resuspended in 100 μL of binding buffer with 5 μL of propidium iodide and 5 μL of FITC annexin V. The cells were gently mixed on a vortexer and incubated for 15 min at room temperature (20–25 °C) in the dark. Afterwards, 400 μL of binding buffer was added to each tube and the cells were analyzed by flow cytometry on a BD LSRFortessa cytometer using BD FACSDiva Software (BD Biosciences) and Flowjo Software 10 (Flowjo LCC, Ashland, OR, USA). Ten thousand events were evaluated per experiment and cellular debris was omitted from the analysis.

#### 3.3.6. Internucleosomal DNA Fragmentation and Cell Cycle Distribution

Cells were harvested in a lysis solution containing 0.1% triton X-100 (Sigma Chemical Co., St. Louis, MO, USA), 2 µg/mL propidium iodide (Sigma Chemical Co.), 0.1% sodium citrate and 100 µg/mL RNAse (Sigma Chemical Co.). Cell fluorescence was determined by flow cytometry on a BD LSRFortessa cytometer using BD FACSDiva Software (BD Biosciences) and Flowjo Software 10. Ten thousand events were evaluated per experiment and cellular debris was omitted from the analysis.

#### 3.3.7. Statistical Analysis

Data are presented as the mean ± SEM or the IC_50_ value, and the 95% confidence intervals were obtained by nonlinear regression from at least three independent experiments performed in duplicate or triplicate. Differences among experimental groups were compared by one-way analysis of variance (ANOVA) followed by the Newman-Keuls test (*p* < 0.05). All analyses were carried out using the GRAPHPAD program (Intuitive Software for Science, San Diego, CA, USA).

## 4. Conclusions

In conclusion, 19 alkaloids were isolated from the stem of *X. laevigata*, of which lanuginosine, (+)-xylopine and (+)-norglaucine had the most potent cytotoxic activity. Lanuginosine and (+)-xylopine were able to induce apoptosis. In addition, (+)-xylopine treatment caused G_2_/M cycle arrest in HepG2 cells. These data suggest that *X. laevigata* is a potential source for cytotoxic alkaloids.

## Figures and Tables

**Table 1:** Cytotoxic activity of the alkaloids from the stem of *Xylopia laevigata.*

Alkaloids	IC_50_ in μg/mL (µM)				
	B16-F10	HepG2	HL60	K562	PBMC
**(−)-Roemerine**	NA	NA	NA	NA	NA
**(+)-Anonaine**	18.80 (70.66)	14.04 (52.77)	10.09 (37.92)	10.62 (39.91)	NA
	15.53–22.76	12.19–16.16	7.94–12.82	8.96–12.58	
**Lanuginosine**	8.46 (27.63)	3.89 (12.70)	7.81 (25.51)	6.61 (21.59)	24.53 (80.11)
	7.40–9.68	3.23–4.69	7.32–8.33	5.69–7.68	16.98–35.43
**(+)-Glaucine**	NA	NA	NA	NA	NA
**(+)-Xylopine**	3.77 (12.73)	1.87 (6.31)	1.87 (6.31)	3.12 (10.53)	4.08 (13.77)
	3.39–4.19	1.56–2.23	1.67–2.10	2.85–3.41	2.17–7.66
**Oxoglaucine**	19.14 (54.36)	NA	5.90 (16.76)	12.48 (35.45)	10.25 (29.11)
	15.19–24.11		4.09–8.52	8.84–17.61	7.96–13.20
**(+)-Norglaucine**	8.48 (24.77)	3.78 (11.04)	6.84 (19.98)	7.84 (22.90)	6.70 (19.57)
	7.62–9.44	3.11–4.61	6.25–7.48	6.78–9.06	3.74–12.00
**(−)-Xylopinine**	NA	NA	NA	NA	NA
**(+)-Norpurpureine**	21.08 (56.61)	NA	10.11 (27.15)	16.72 (44.90)	17.94 (48.18)
	16.74–26.53		5.75–17.81	11.73–23.84	13.23–24.33
**(+)-*N*-Methyllaurotetanine**	NA	NA	NA	NA	NA
**(+)-Norpredicentrine**	NA	NA	NA	NA	NA
**(+)-Discretine**	16.15 (47.20)	7.89 (23.06)	12.97 (37.91)	14.85 (43.40)	NA
	14.35–18.17	5.83–10.68	10.75–15.66	11.89–18.54	
**(+)-Calycinine**	22.17 (71.03)	NA	18.59 (59.56)	NA	NA
	12.67–38.81		13.45–25.69		
**(+)-Laurotetanine**	NA	NA	NA	NA	NA
**(+)-Reticuline**	NA	15.35 (46.47)	23.81 (72.09)	NA	NA
		12.45–18.92	21.44–26.45		
**(−)-Corytenchine**	NA	NA	NA	NA	NA
**(+)-Discretamine**	18.80 (57.29)	14.04 (42.79)	10.09 (30.75)	10.62 (32.36)	NA
	15.53–22.76	12.19–16.16	7.94–12.82	8.96–12.58	
**(+)-Flavinantine**	NA	NA	NA	NA	NA
**Doxorubicin**	0.08 (0.15)	0.08 (0.15)	0.09 (0.17)	0.15 (0.28)	2.47 (4.54)
	0.05–0.14	0.06–0.10	0.06–0.12	0.08–0.31	1.80–3.39

Data are presented as IC_50_ values in μg/mL (μM) and their 95% confidence interval obtained by nonlinear regression from three independent experiments performed in duplicate, measured using an Alamar Blue assay after 72 h of incubation. Doxorubicin was used as the positive control. NA: not active instead (IC_50_ > 25 μg/mL).

**Table 2:** Effect of (+)-xylopine on cell cycle distribution of hepatocellular carcinoma (HepG2) cells after 24 h of incubation.

Drug	Concentration (µg/mL)	Cell Cycle Phases (%)		
		G_1_	S	G_2_/M
NC	-	51.50 ± 4.02	16.11 ± 1.89	19.93 ± 6.12
PC	1	21.44 ± 4.07 *	12.00 ± 1.43 *	61.94 ± 5.52 *
(+)-xylopine	5	28.05 ± 3.15 *	10.80 ± 1.50 *	64.16 ± 2.56 *
	10	20.43 ± 3.06 *	11.56 ± 1,78 *	39.96 ± 9.59 *

The data are presented as the mean values ± S.E.M. from at least three independent experiments performed in duplicate. Negative control (NC) was treated with the vehicle (0.2% DMSO) used for diluting the tested substance. Doxorubicin (1 μg/mL) was used as the positive control (PC). Ten thousand events were evaluated per experiment and cellular debris was omitted from the analysis. * *p* < 0.05 compared to negative control by ANOVA followed by the Student-Newman-Keuls test.

## Supplementary Materials

Supplementary materials can be accessed at: http://www.mdpi.com/1420-3049/21/7/890/s1.
