# Supplementary material for: Cytotoxic Alkaloids from the Stem of Xylopia laevigata
Source: Molecules. 2016 Jul 8;21(7):890. doi: 10.3390/molecules21070890 (PMC6273342; doi:10.3390/molecules21070890)
Supplement: Supplementary file 1 [file molecules-21-00890-s001.pdf]

Supplementary Materials: Cytotoxic Alkaloid from the Stem of *Xylopia laevigata*

Leociley R. A. Menezes, Cinara O. D´Sousa Costa, Ana Carolina B. da C. Rodrigues, Felipe R. do E. Santo, Angelita Nepel, Lívia M. Dutra, Felipe M. A. Silva, Milena B. P. Soares, Andersson Barison, Emmanoel V. Costa and Daniel P. Bezerra

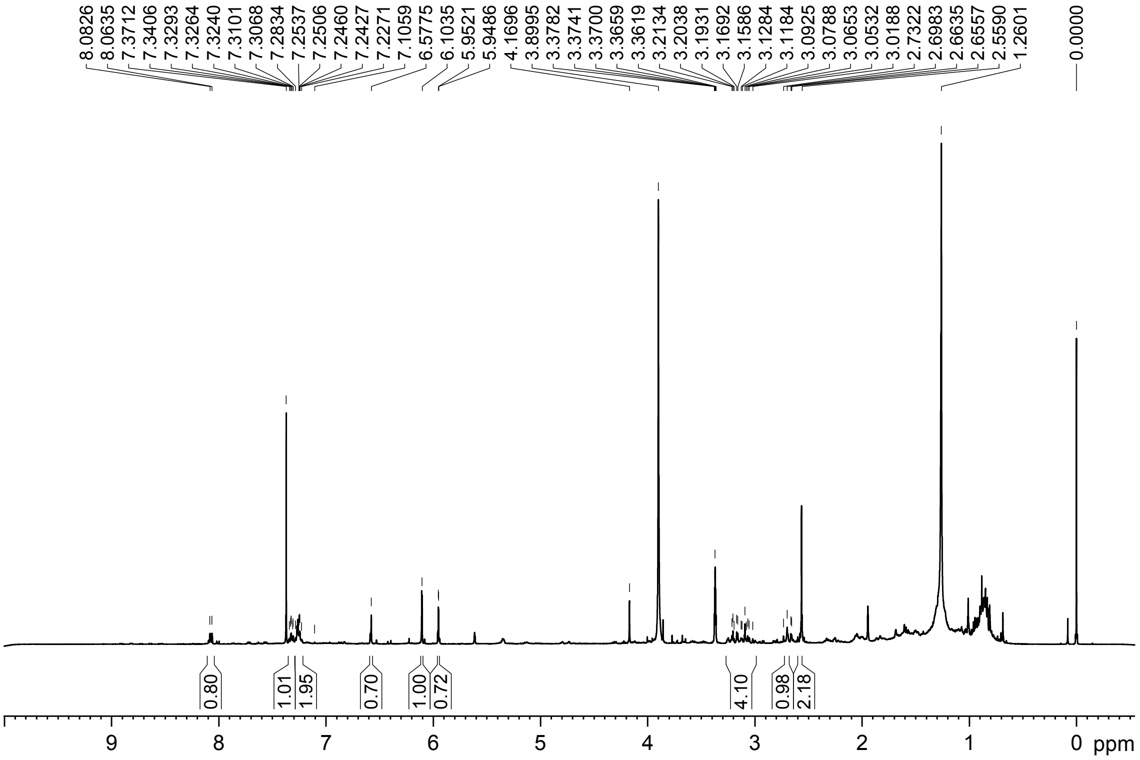

Figure S1. ^{1}H NMR spectrum of alkaloid **(-)-roemerine** ($CDCl_3$ + drops of CD_3OD at 400 MHz).

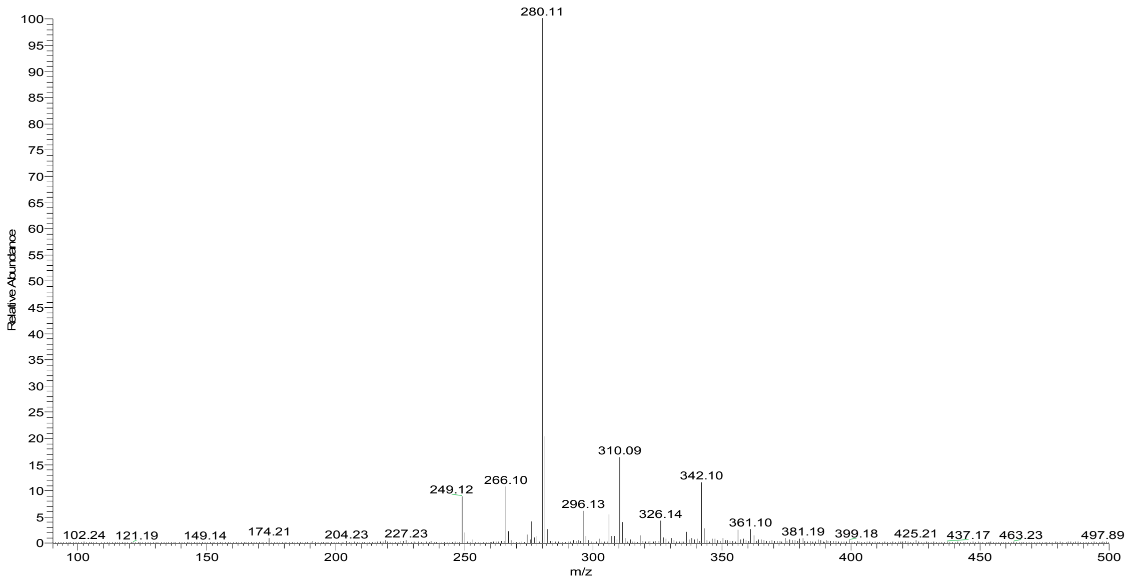

Figure S2. MS of alkaloid **(−)-roemerine** (*m*/*z* 280.11 $[M + H]^+$).

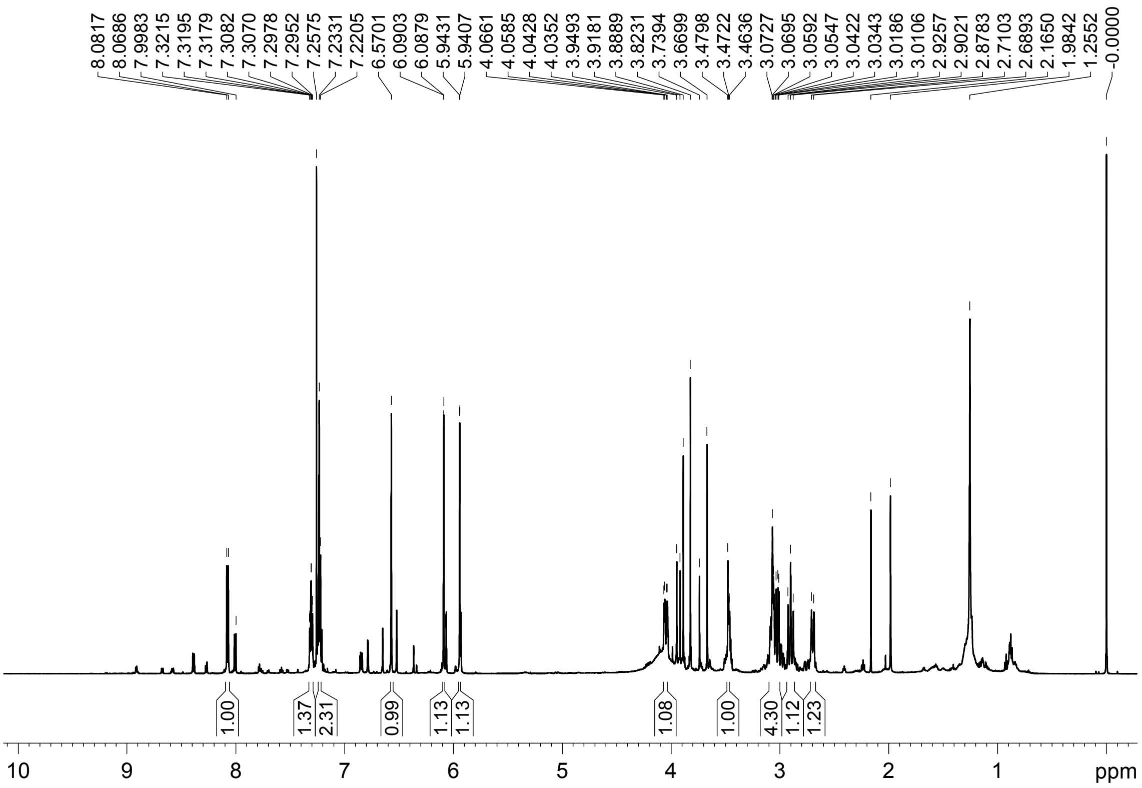

Figure S3. ^{1}H NMR spectrum of alkaloid **(+)-anonaine** ($CDCl_3$ at 400 MHz).

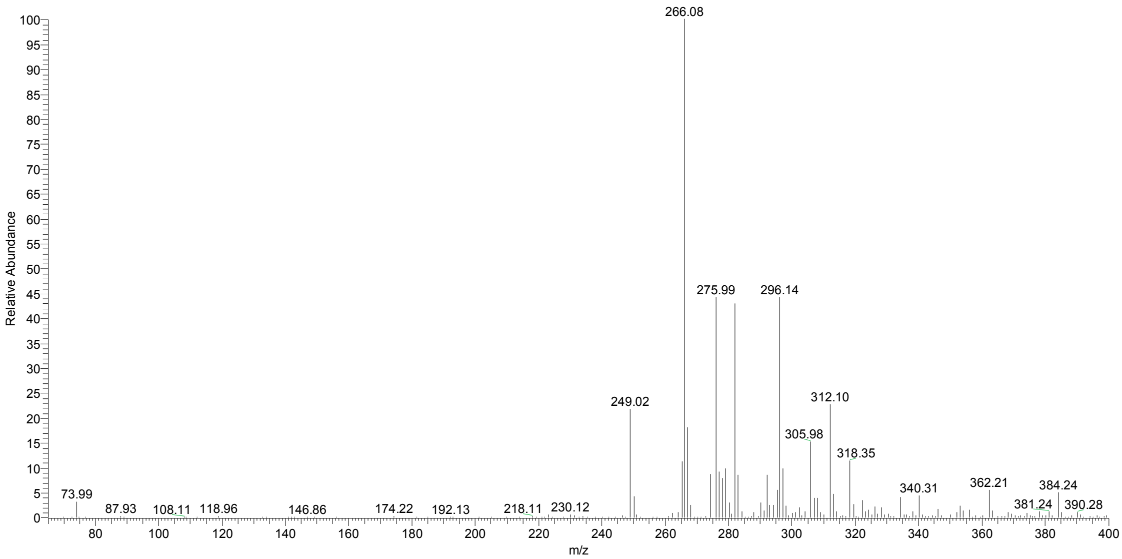

Figure S4. MS of alkaloid **(+)-anonaine** (*m*/*z* 266.08 [M + H]$^+$).

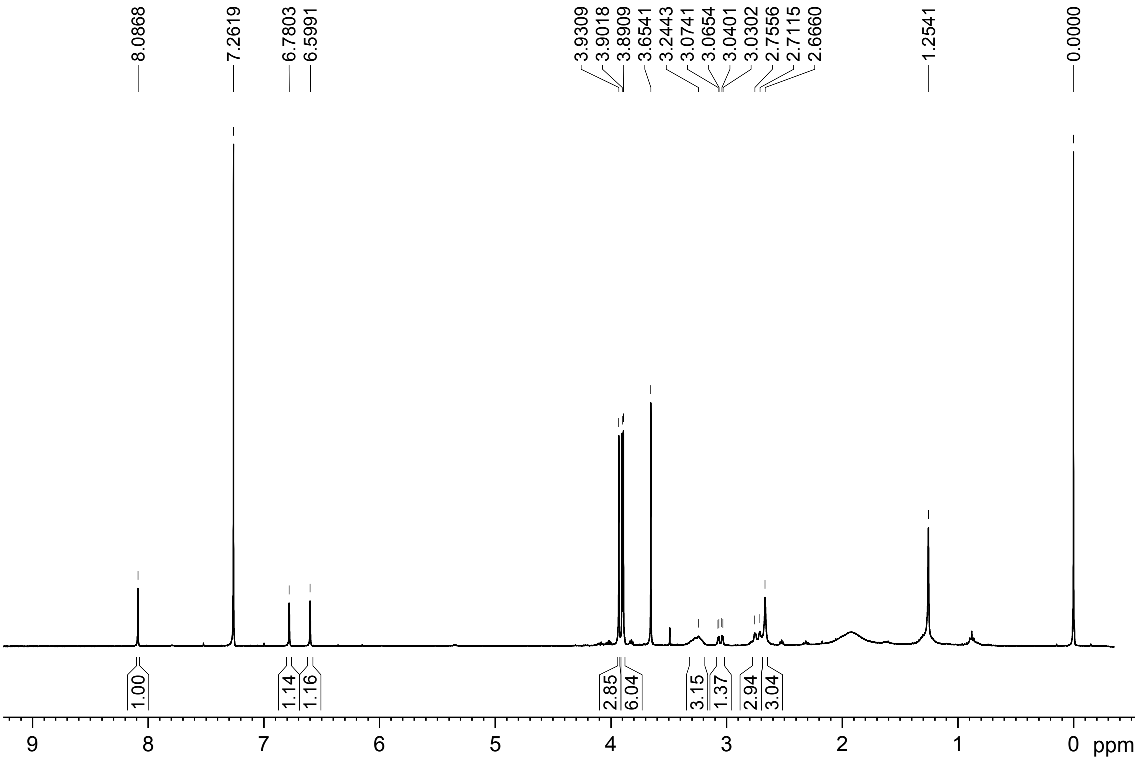

Figure S5. ^{1}H NMR spectrum of alkaloid **(+)-glaucine** ($CDCl_3$ + drops of CD_3OD at 400 MHz).

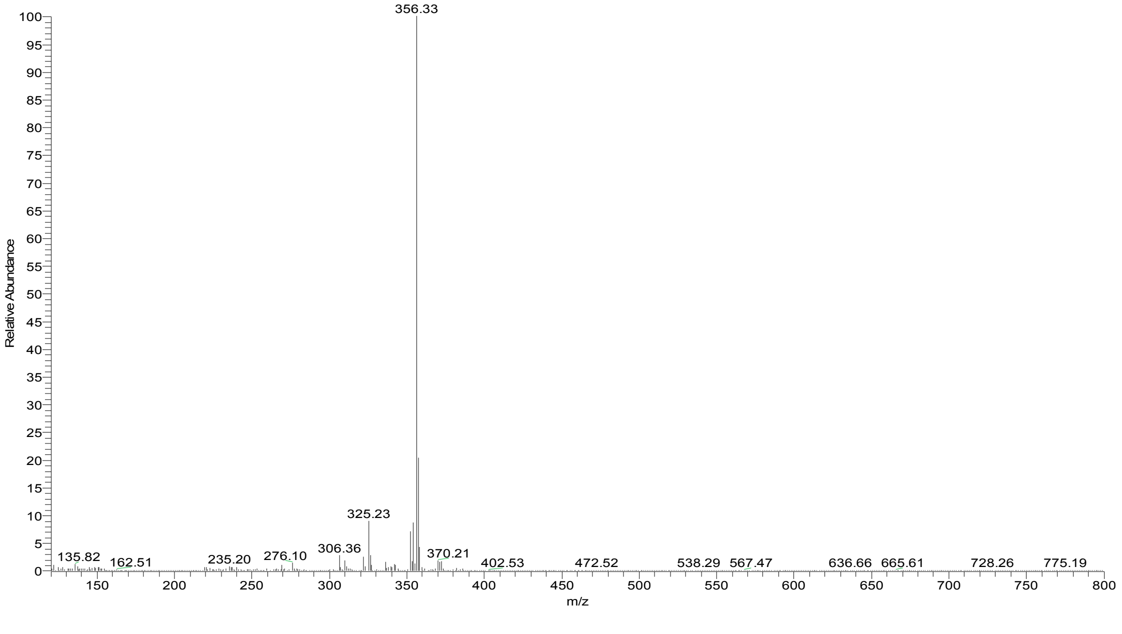

Figure S6. MS of alkaloid **(+)-glaucine** (*m*/*z* 356.33 $[M + H]^+$).

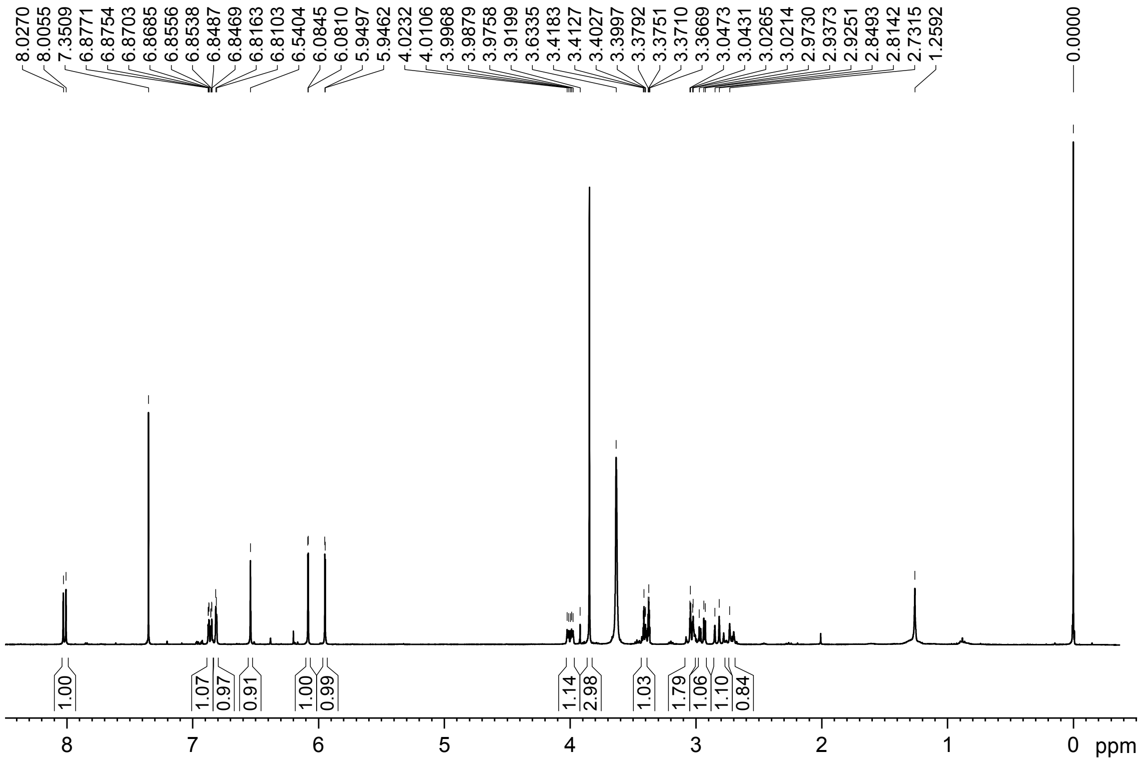

Figure S7. ^{1}H NMR spectrum of alkaloid **(+)-xylopine** ($CDCl_3$ + drops of CD_3OD at 400 MHz).

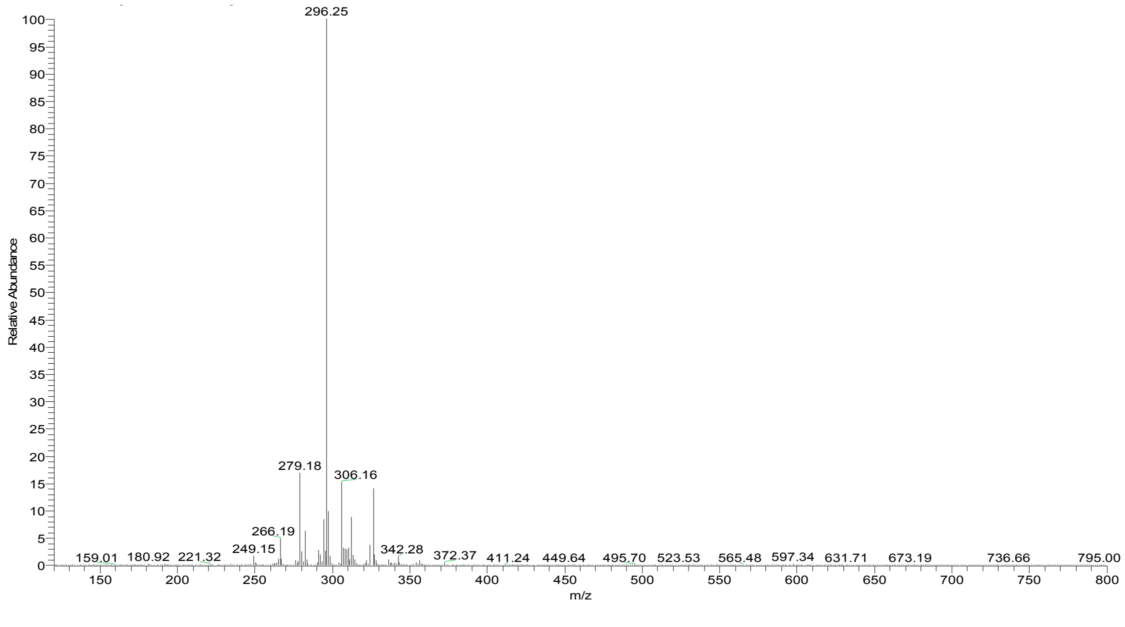

Figure S8. MS of alkaloid **(+)-xylopine** (*m*/*z* 296.25 $[M + H]^+$).

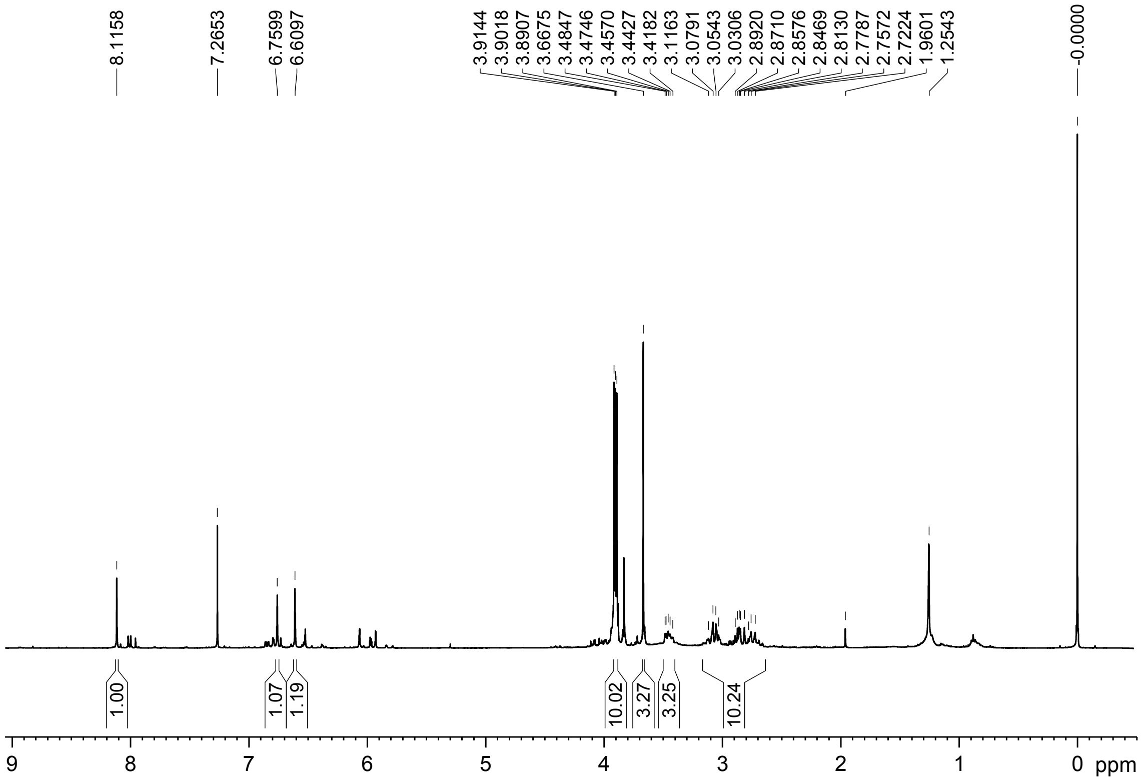

Figure S9. ^{1}H NMR spectrum of alkaloid **(+)-norglaucine** ($CDCl_3$ + drops of CD_3OD at 400 MHz).

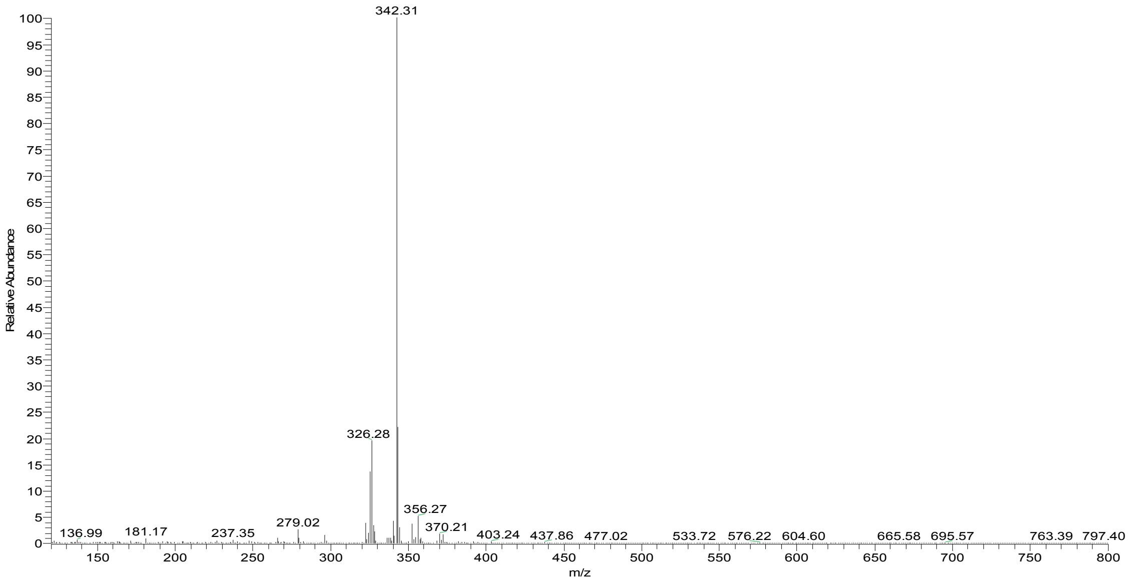

Figure S10. MS of alkaloid **(+)-norglaucine** (*m*/*z* 342.31 [M + H]$^+$).

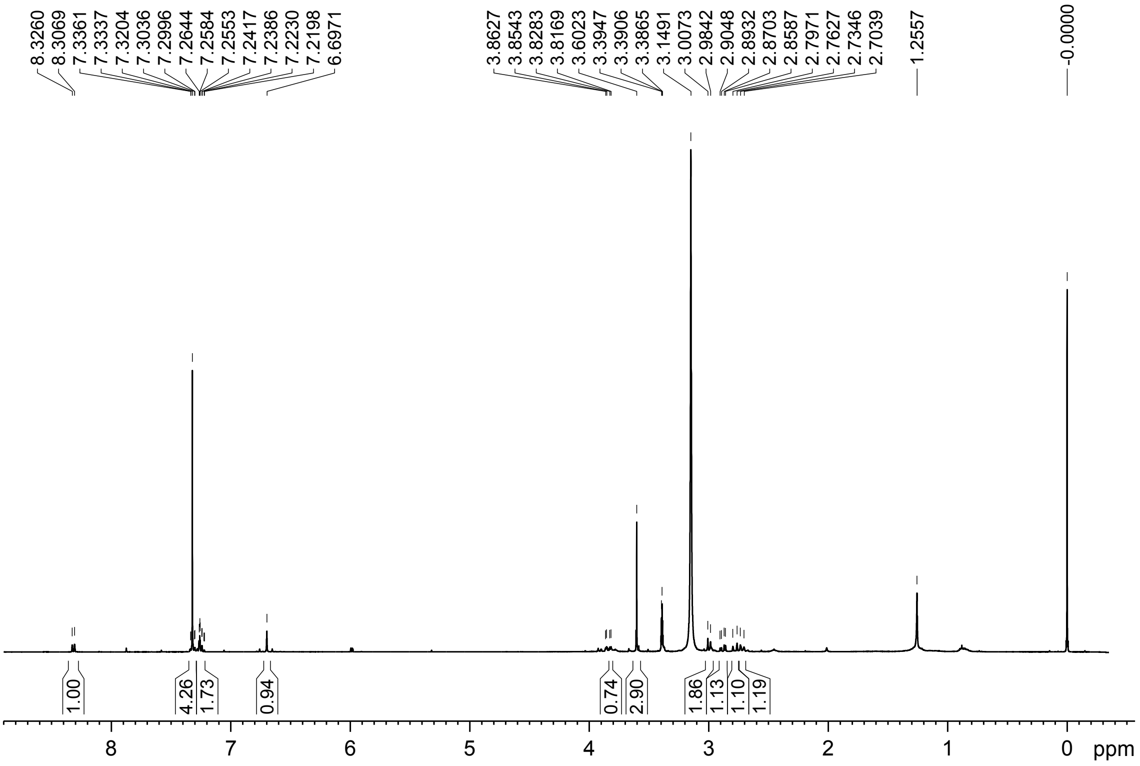

Figure S11. ^{1}H NMR spectrum of alkaloid **asimilobine** ($CDCl_3$ + drops of CD_3OD at 400 MHz).

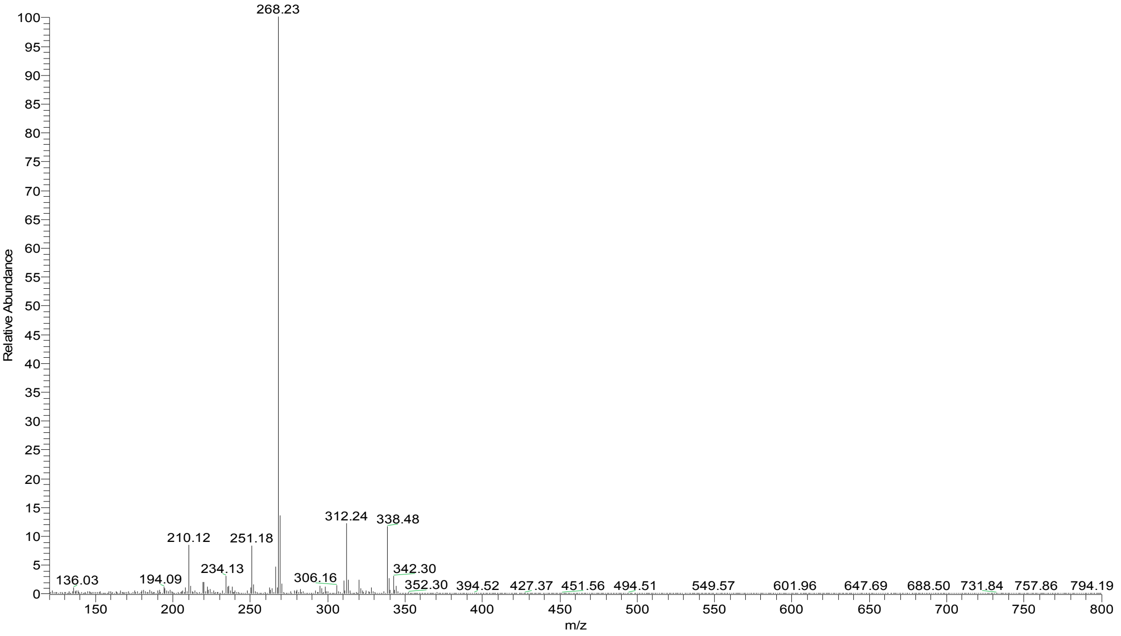

Figure S12. MS of alkaloid **asimilobine** (*m*/*z* 268.23 [M + H]$^+$).

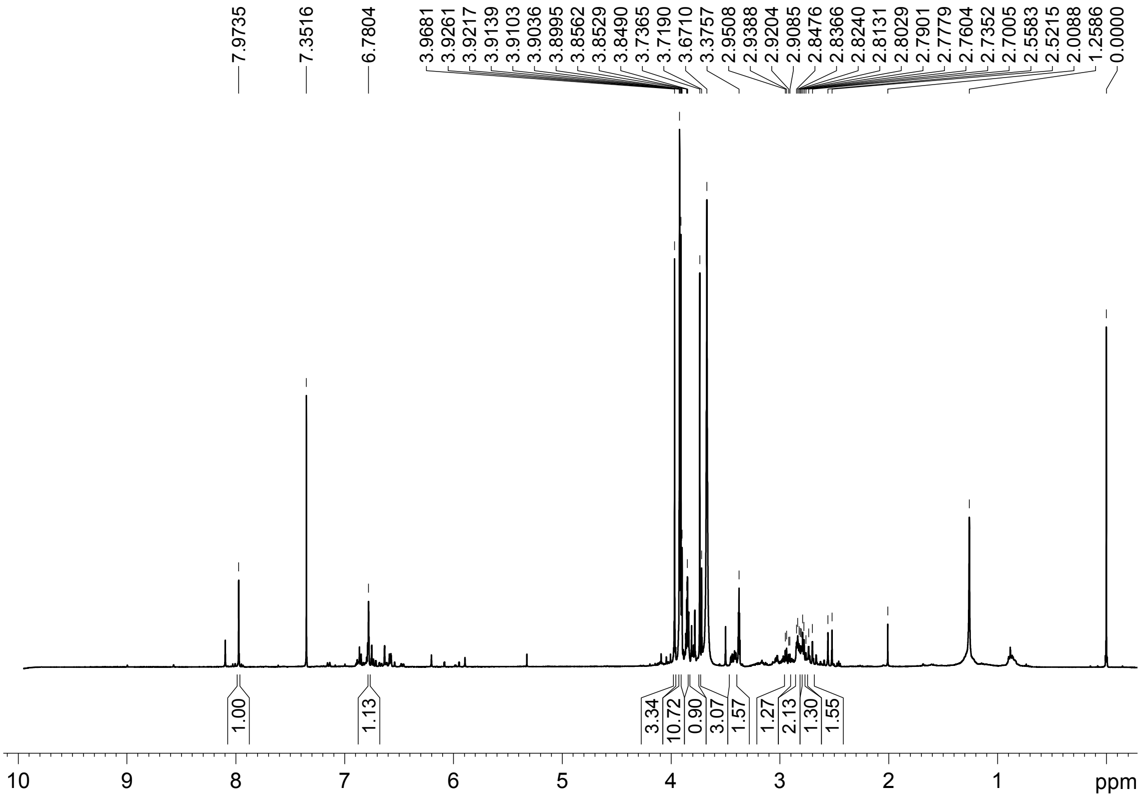

Figure S13. ^{1}H NMR spectrum of alkaloid **(+)-norpurpureine** ($CDCl_3$ + drops of CD_3OD at 400 MHz).

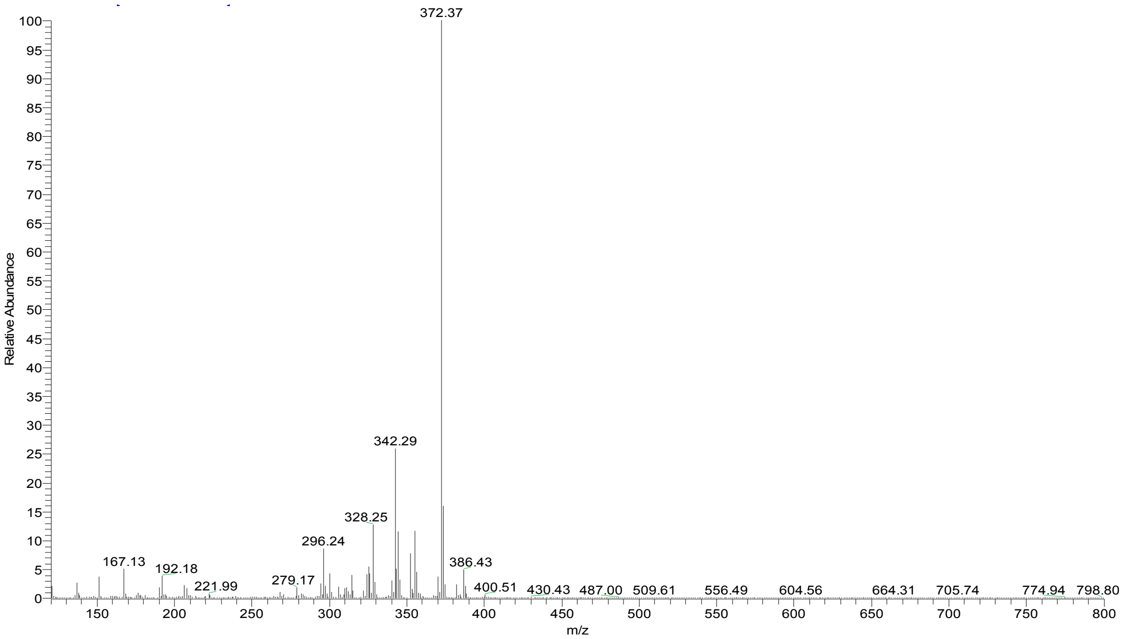

Figure S14. MS of alkaloid **(+)-norpurpureine** (*m*/*z* 372.37 $[M + H]^+$).

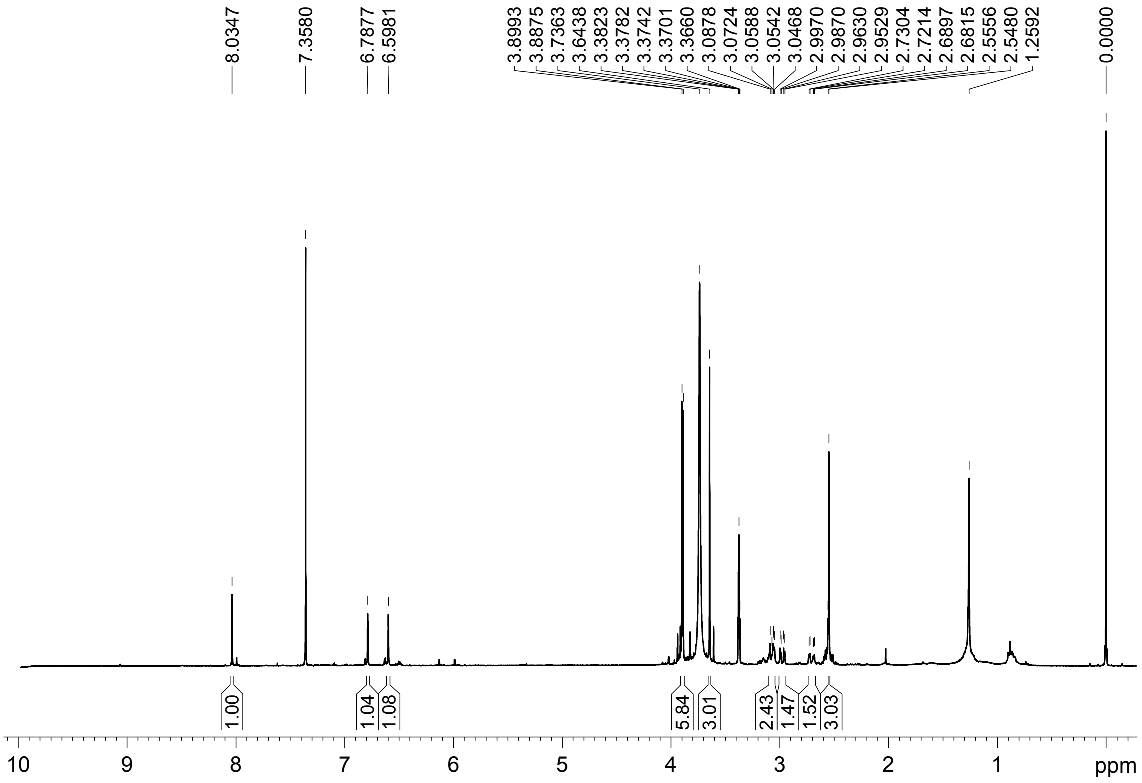

Figure S15. ^{1}H NMR spectrum of alkaloid **(+)-*N*-methyllaurotetanine** ($CDCl_3$ + drops of CD_3OD at 400 MHz).

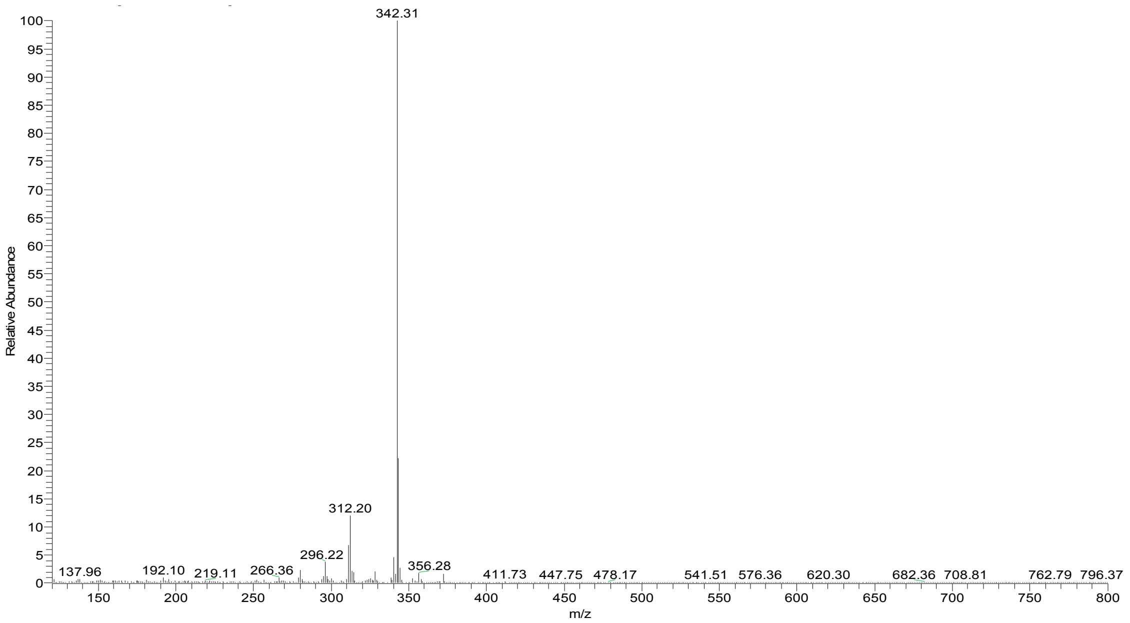

Figure S16. MS of alkaloid **(+)-*N*-methyllaurotetanine** (*m*/*z* 342.31 [M + H]$^+$).

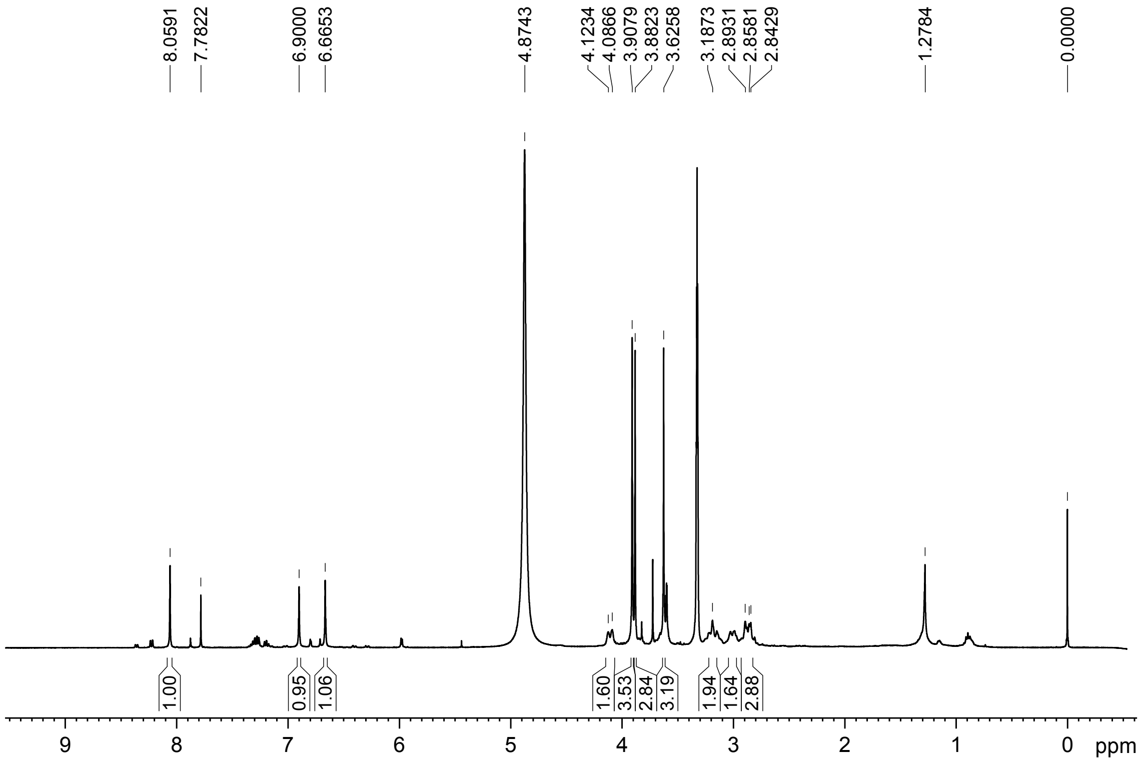

Figure S17. ^{1}H NMR spectrum of alkaloid **(+)-norpredicentrine** (CD_3OD + drops of $CDCl_3$ at 400 MHz).

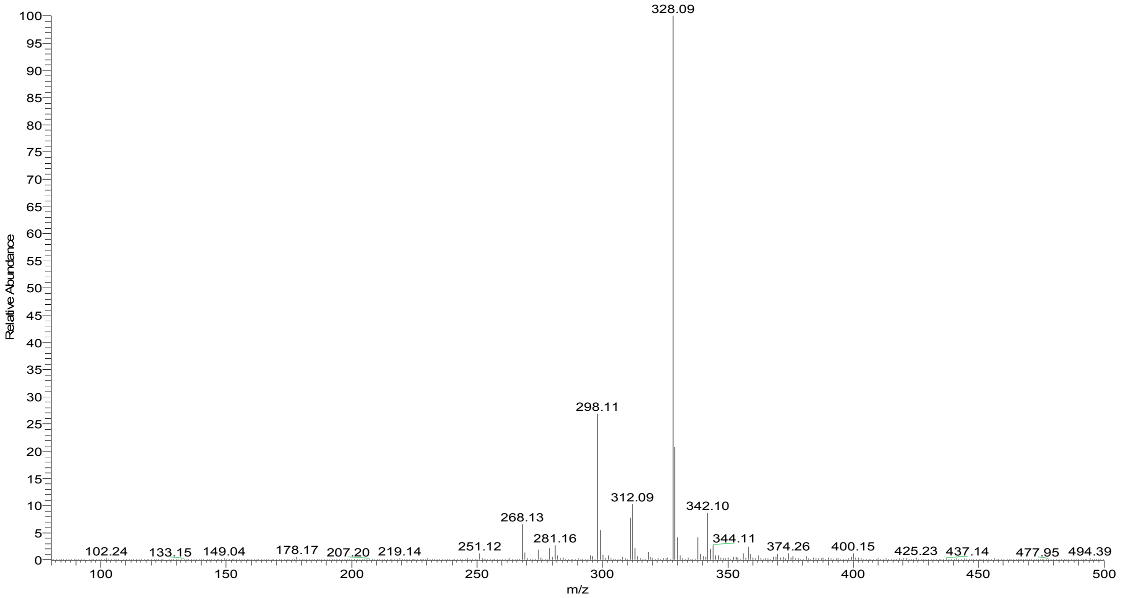

Figure S18. MS of alkaloid **(+)-norpredicentrine** (*m*/*z* 328.09 $[M + H]^+$).

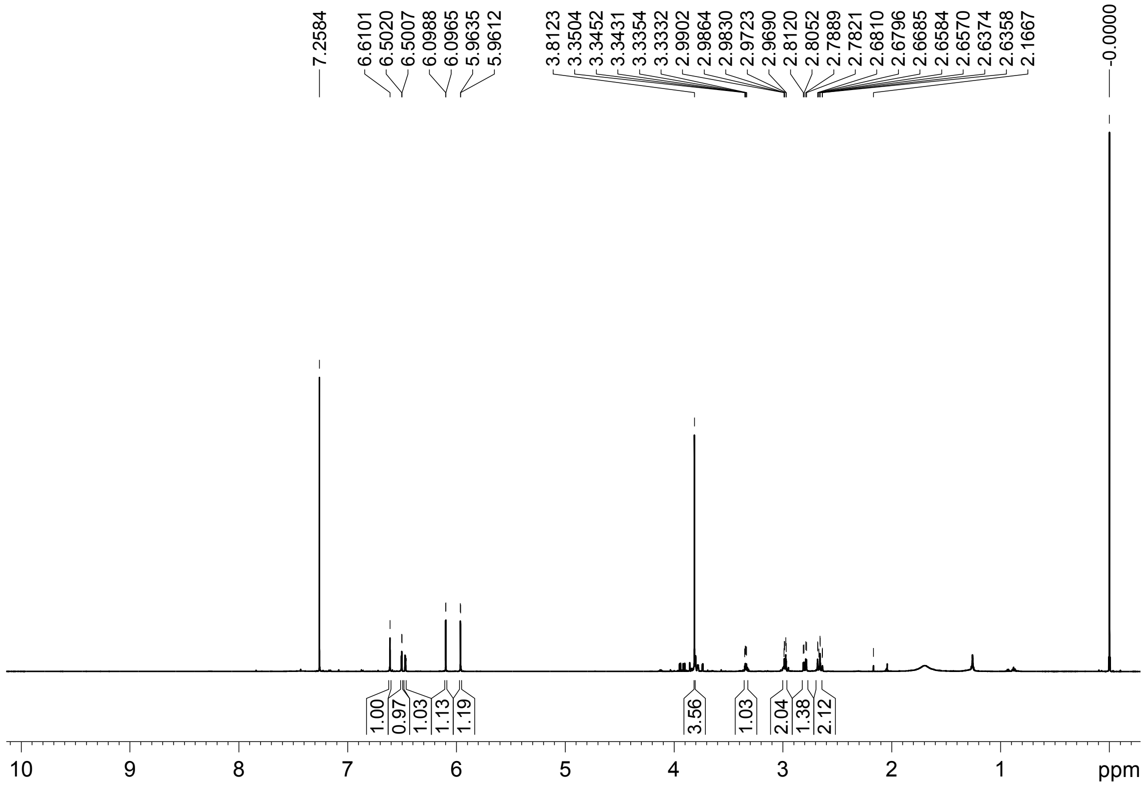

Figure S19. ^{1}H NMR spectrum of alkaloid **(+)-calycinine** ($CDCl_3$ at 400 MHz).

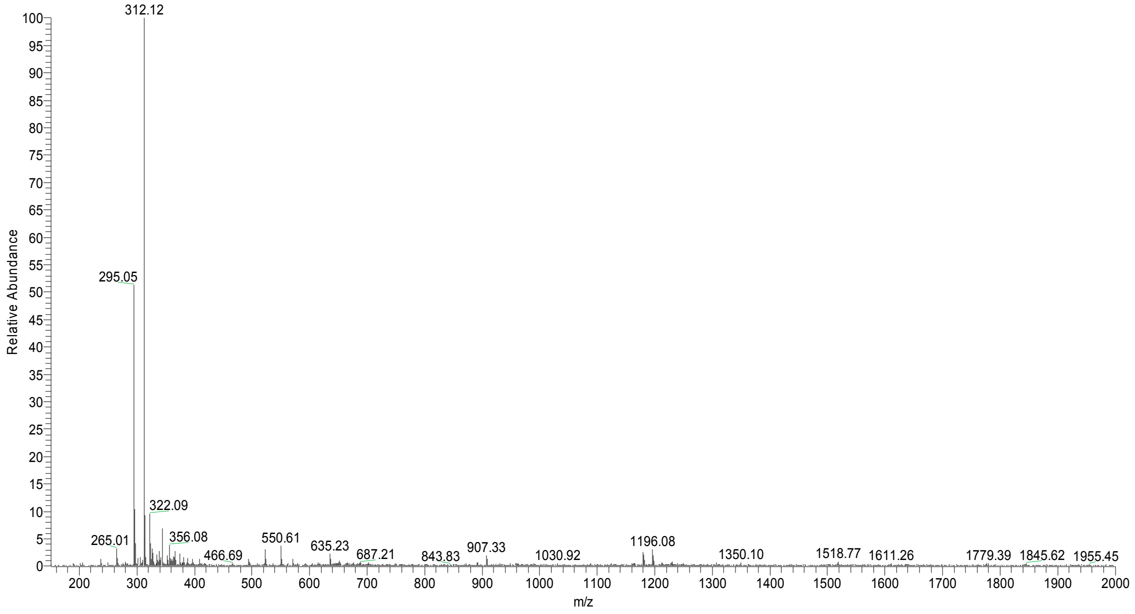

Figure S20. MS of alkaloid **(+)-calycinine** (*m/z* 312.12 [M + H]$^+$).

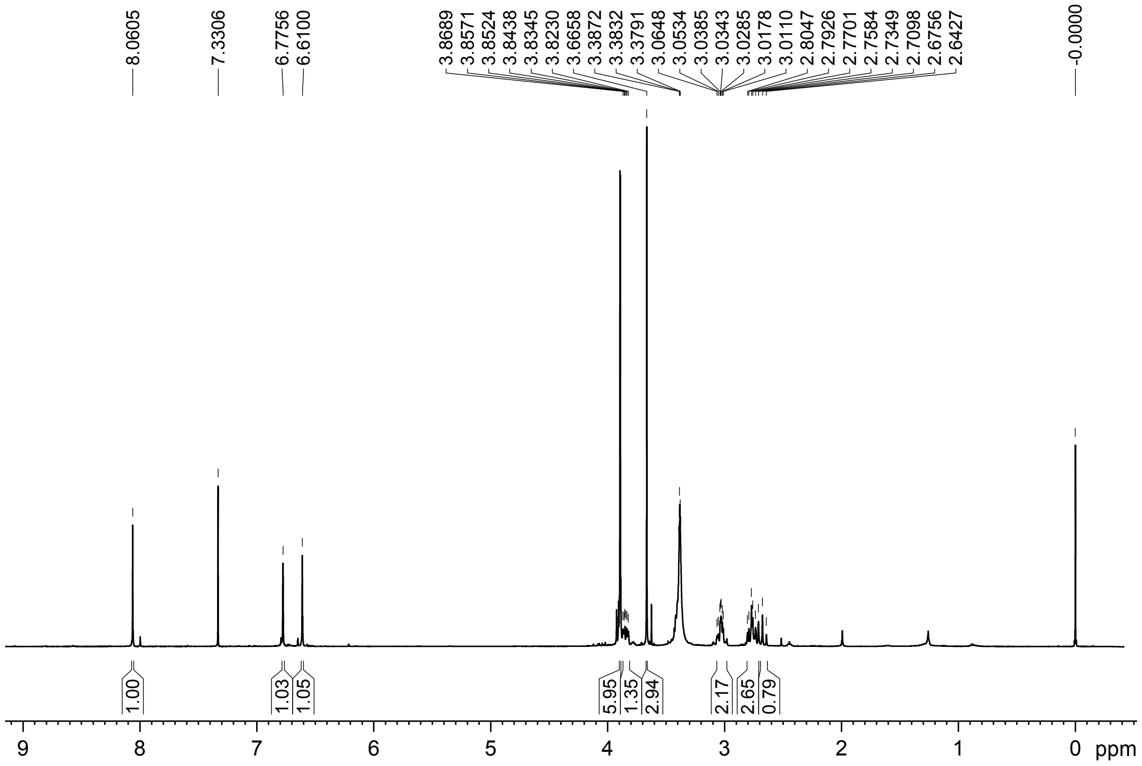

Figure S21. ^{1}H NMR spectrum of alkaloid **(+)-laurotetanine** ($CDCl_3$ + drops of CD_3OD at 400 MHz).

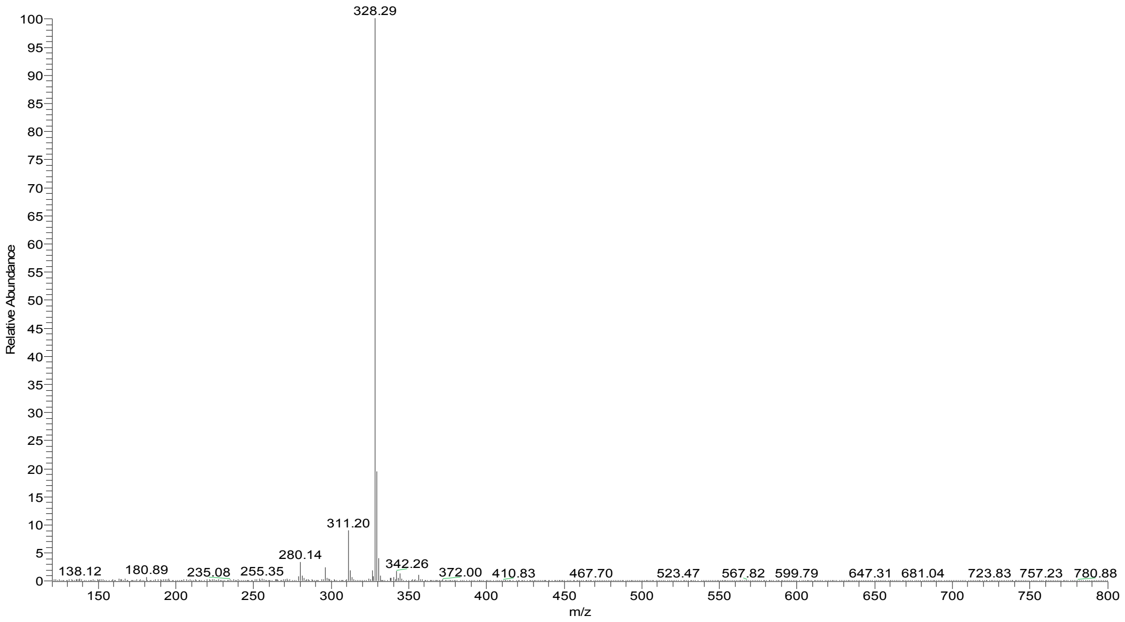

Figure S22. MS of alkaloid **(+)-laurotetanine** (*m*/*z* 328.29 $[M + H]^+$).

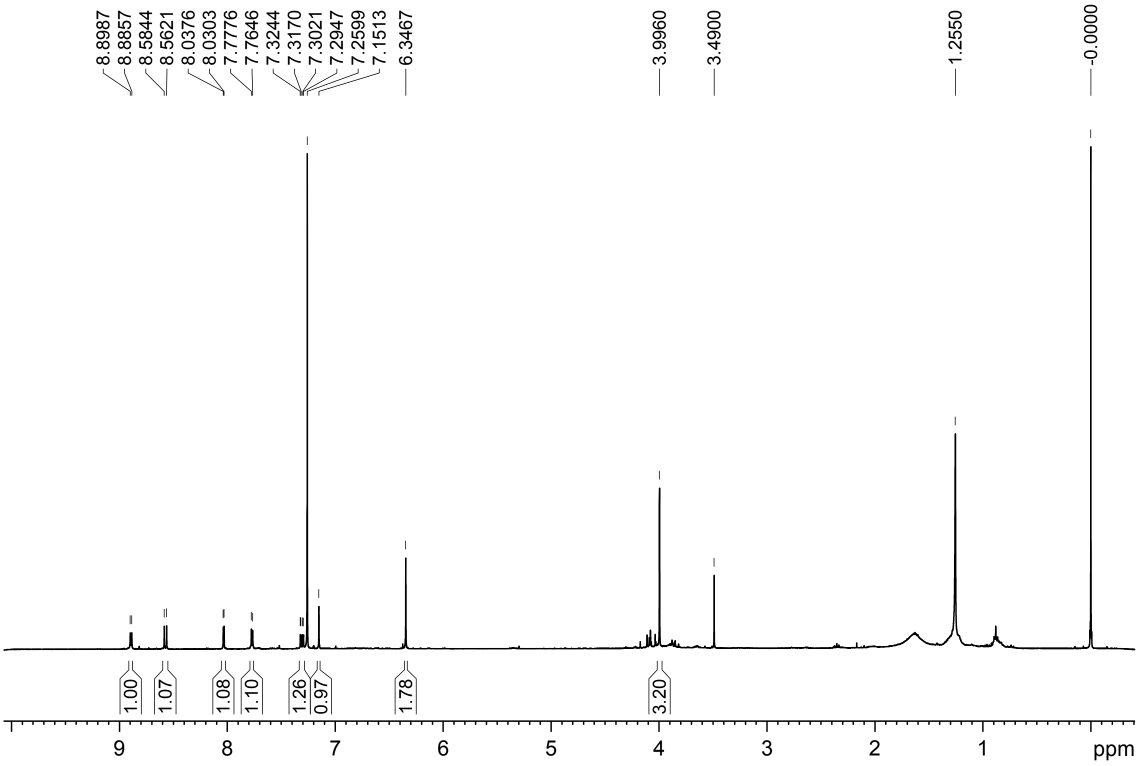

Figure S23. ^{1}H NMR spectrum of alkaloid **lanuginosine** ($CDCl_3$ + drops of CD_3OD at 400 MHz).

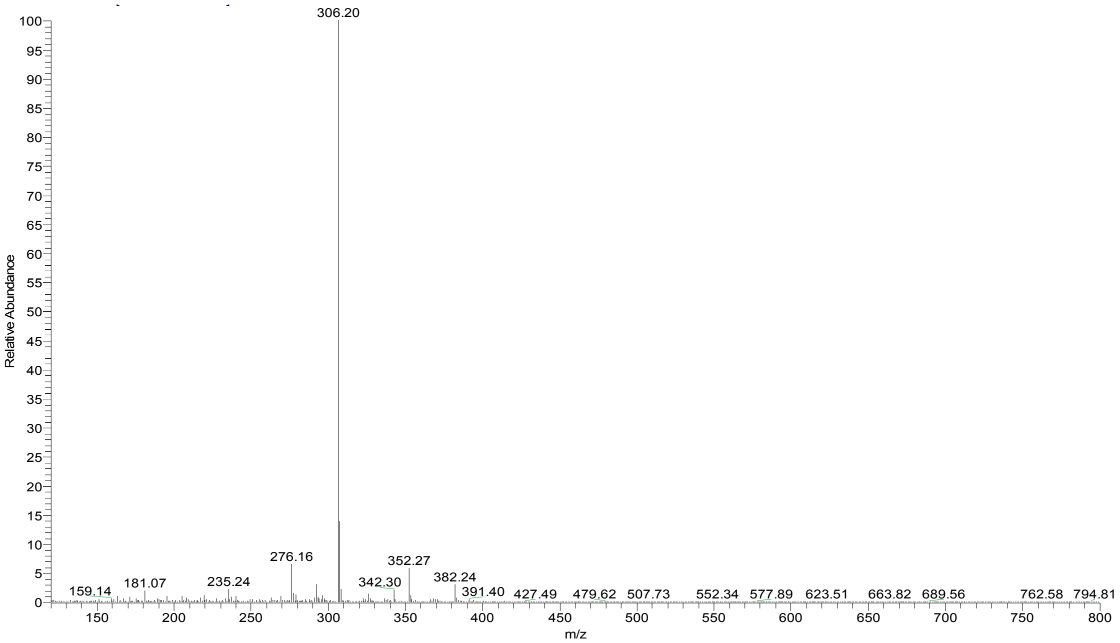

Figure S24. MS of alkaloid **lanuginosine** (*m*/*z* 306.20 [M + H]$^+$).

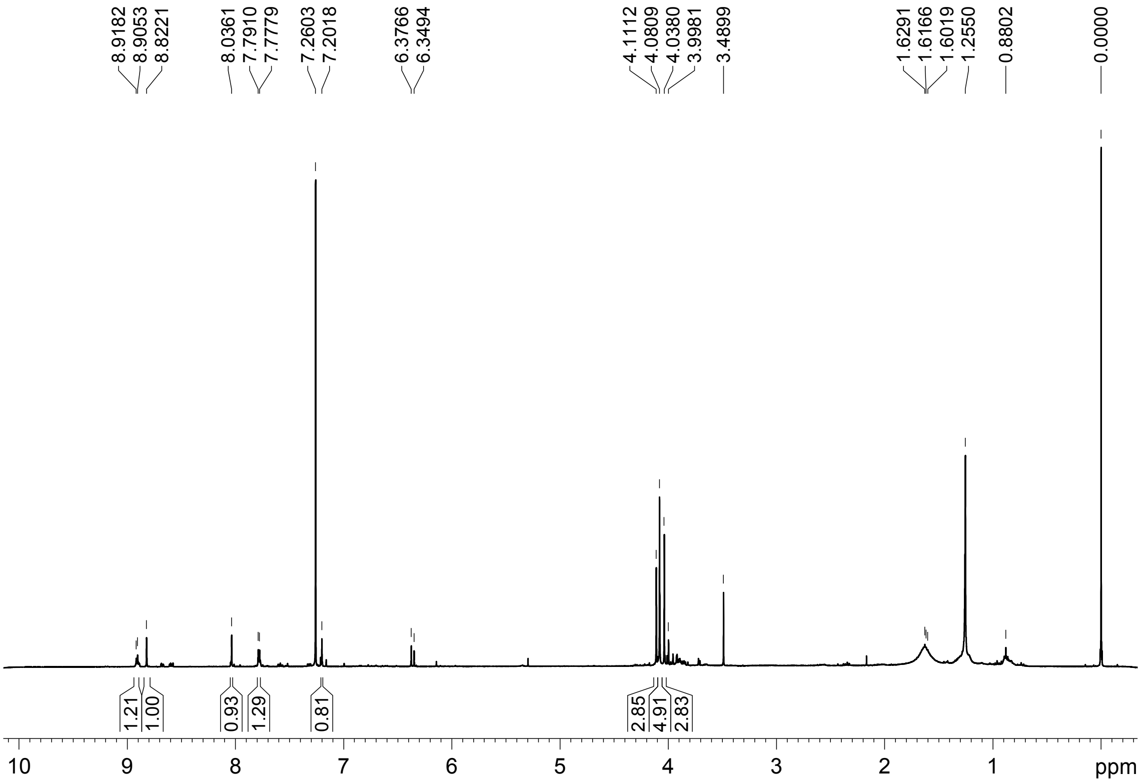

Figure S25. ^{1}H NMR spectrum of alkaloid **oxoglaucine** ($CDCl_3$ + drops of CD_3OD at 400 MHz).

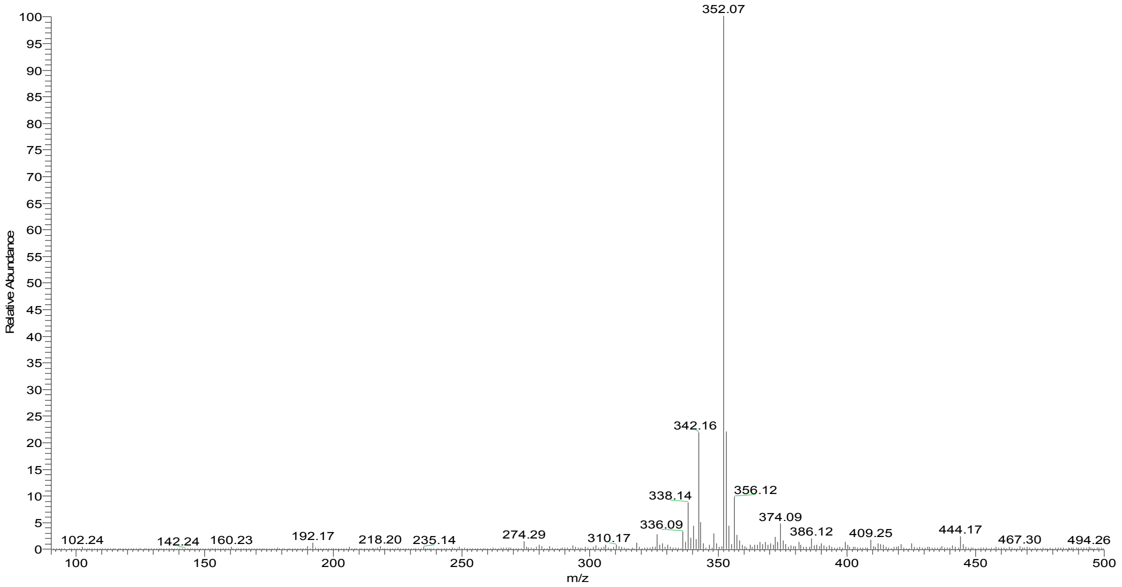

Figure S26. MS of alkaloid **oxoglaucine** (*m*/*z* 352.07 $[M + H]^+$).

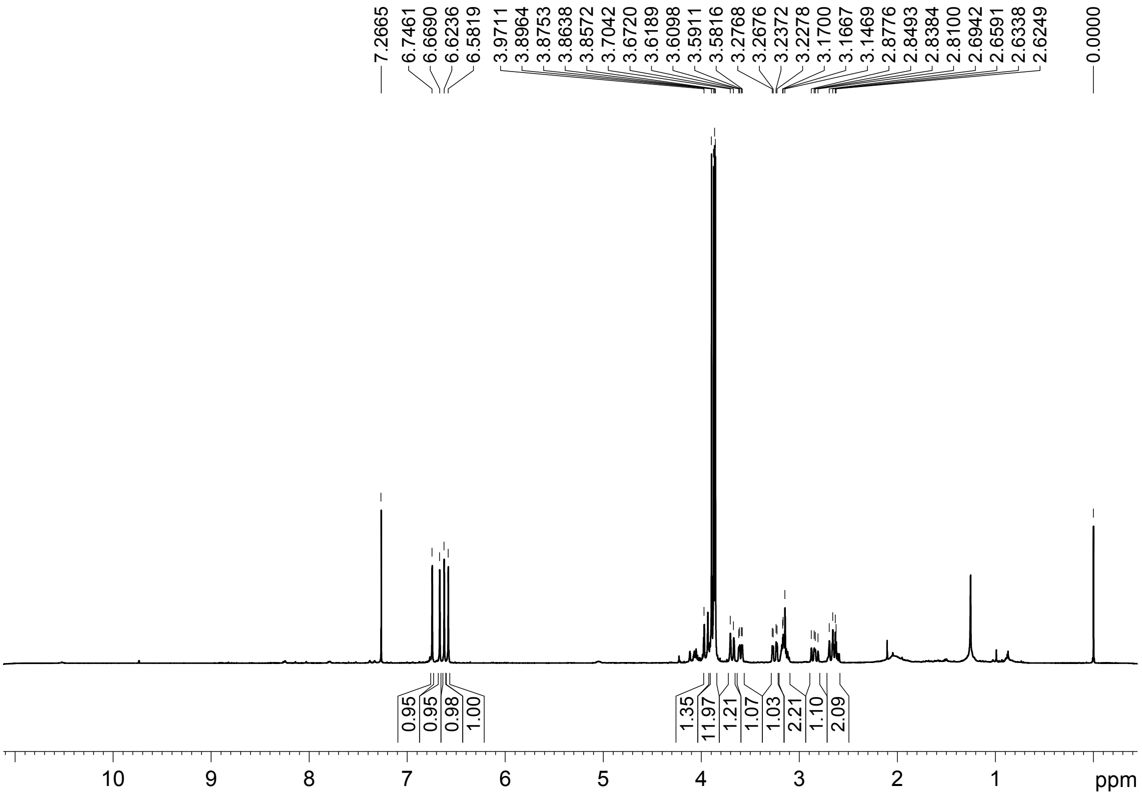

Figure S27. ^{1}H NMR spectrum of alkaloid **(−)-xylopinine** ($CDCl_3$ + drops of CD_3OD at 400 MHz).

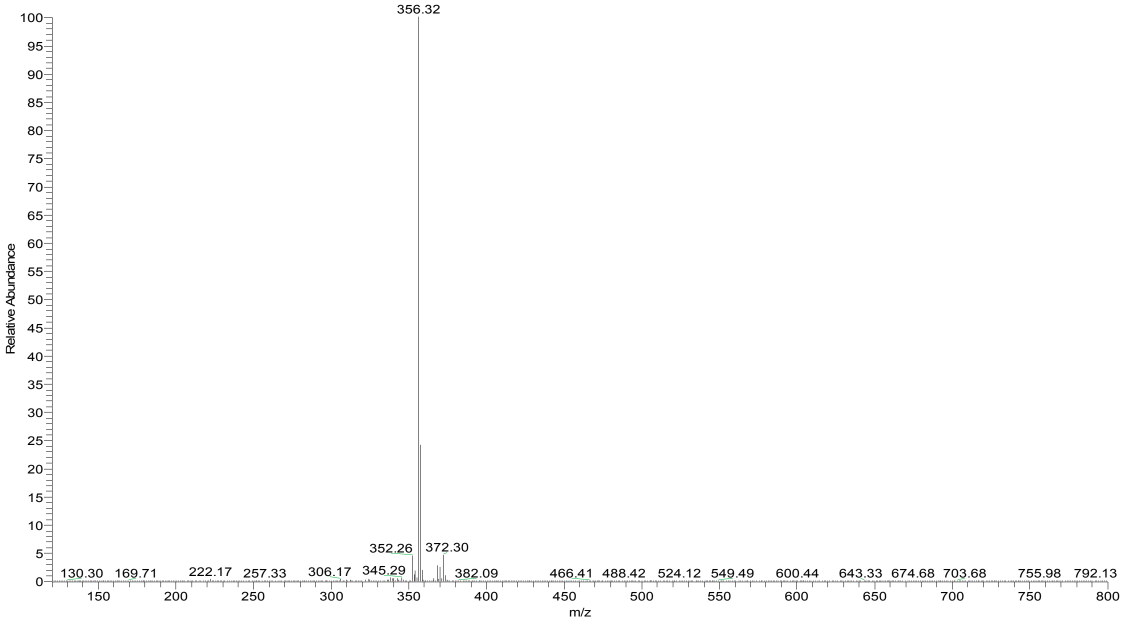

Figure S28. MS of alkaloid **(−)-xylopinine** (*m*/*z* 356.32 [M + H]$^{+}$).

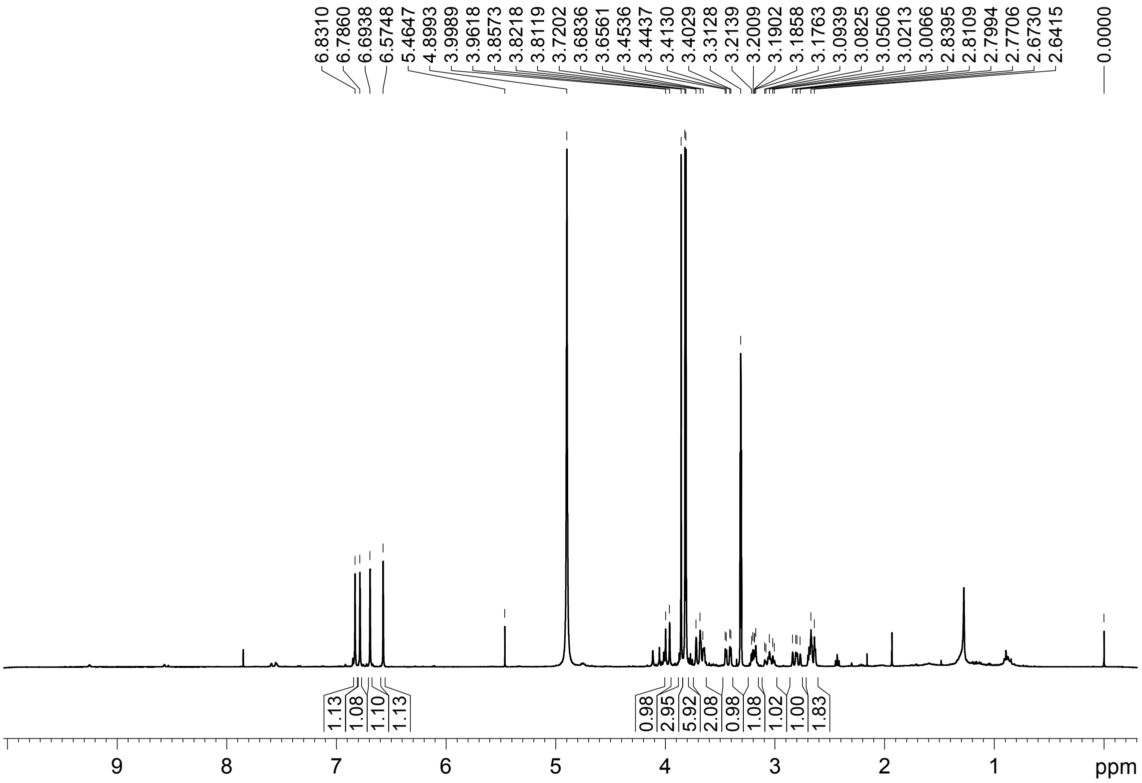

Figure S29. ^{1}H NMR spectrum of alkaloid **(+)-discretine** (CD_3OD + drops of $CDCl_3$ at 400 MHz).

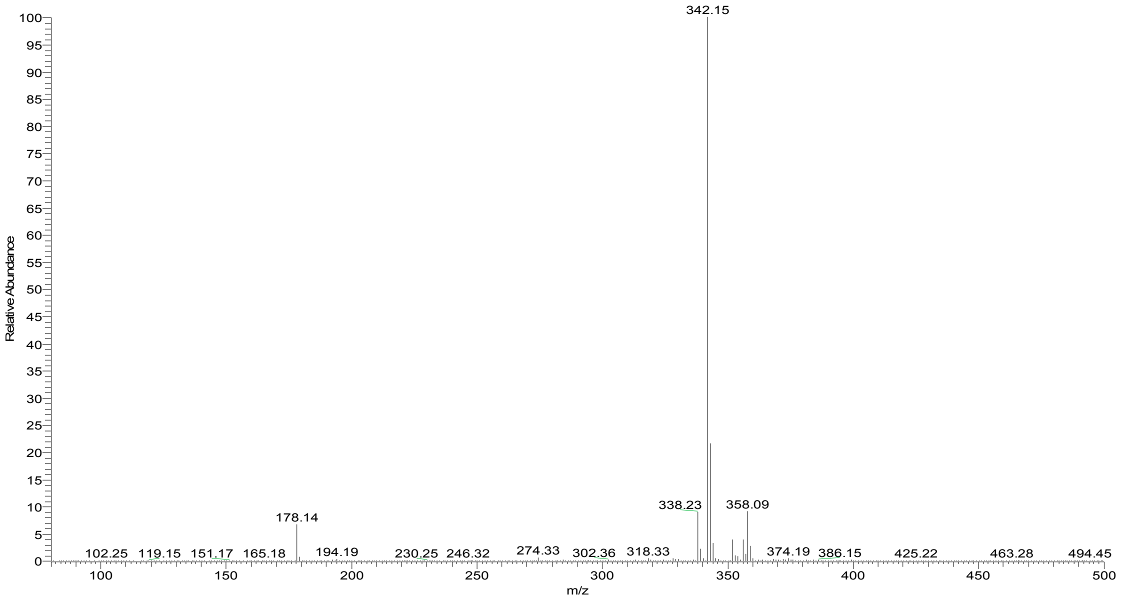

Figure S30. MS of alkaloid **(+)-discretine** (*m*/*z* 342.15 $[M + H]^+$).

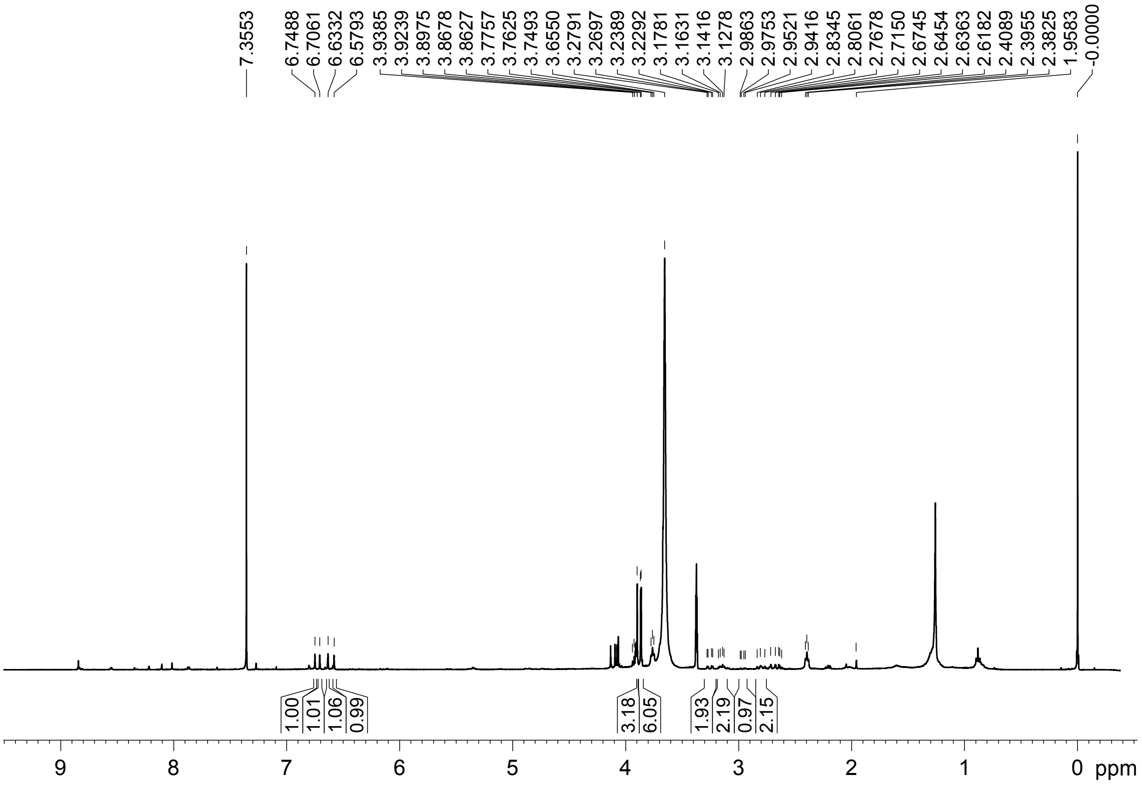

Figure S31. ^{1}H NMR spectrum of alkaloid **(−)-corytenchine** ($CDCl_3$ + drops of CD_3OD at 400 MHz).

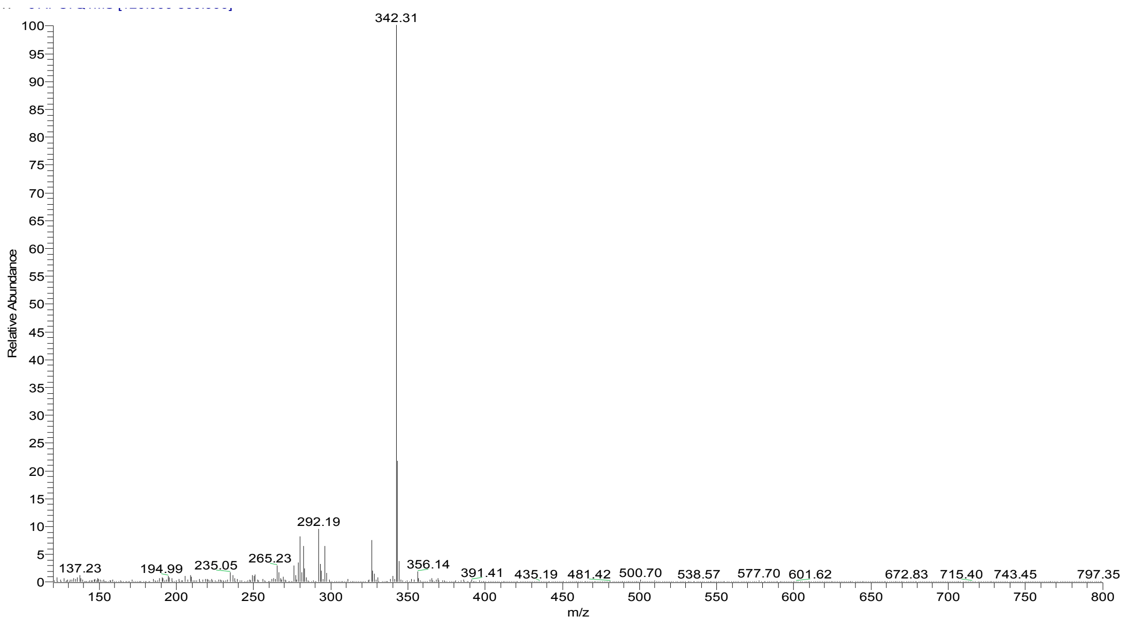

Figure S32. MS of alkaloid **(−)-corytenchine** (*m*/*z* 342.31 [M + H]+).

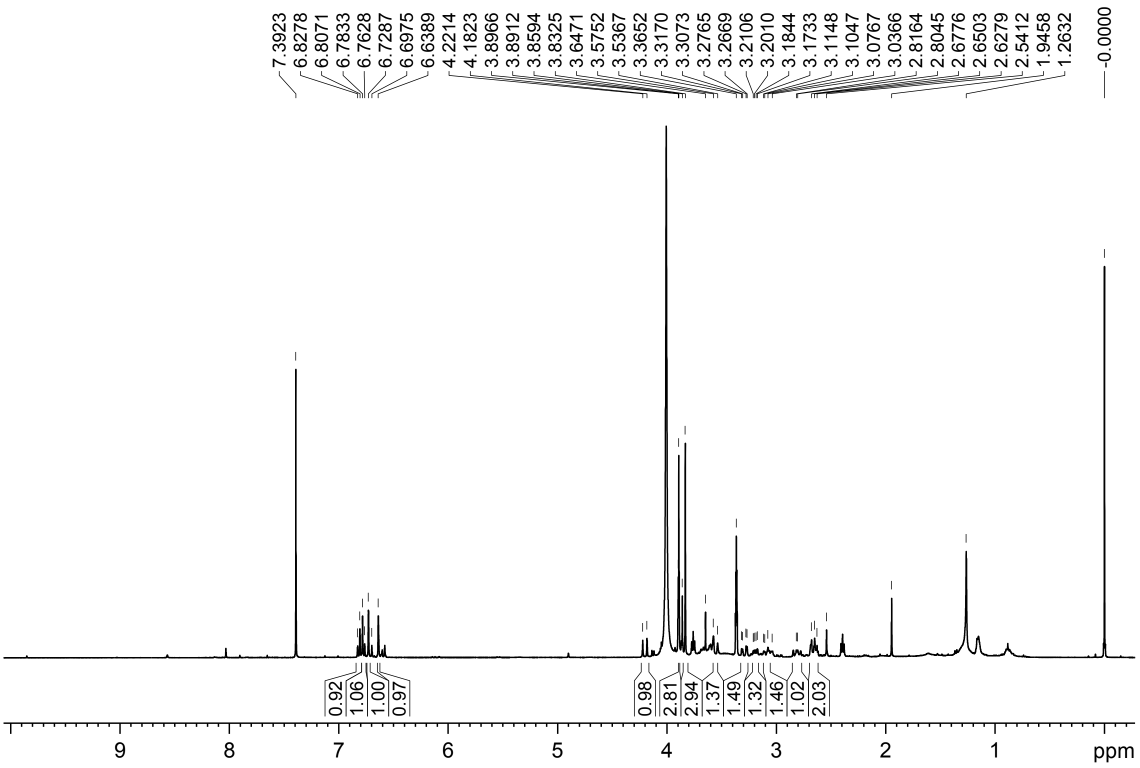

Figure S33. ^{1}H NMR spectrum of alkaloid **(+)-discretamine** ($CDCl_3$ + drops of CD_3OD at 400 MHz).

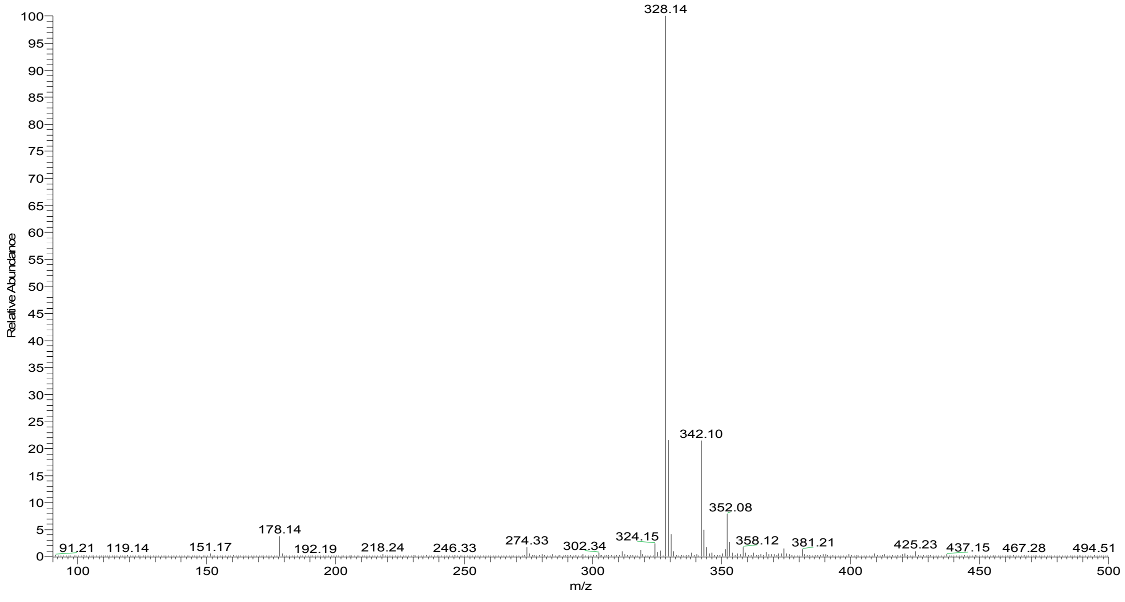

Figure S34. MS of alkaloid **(+)-discretamine** (*m*/*z* 328.14 $[M + H]^+$).

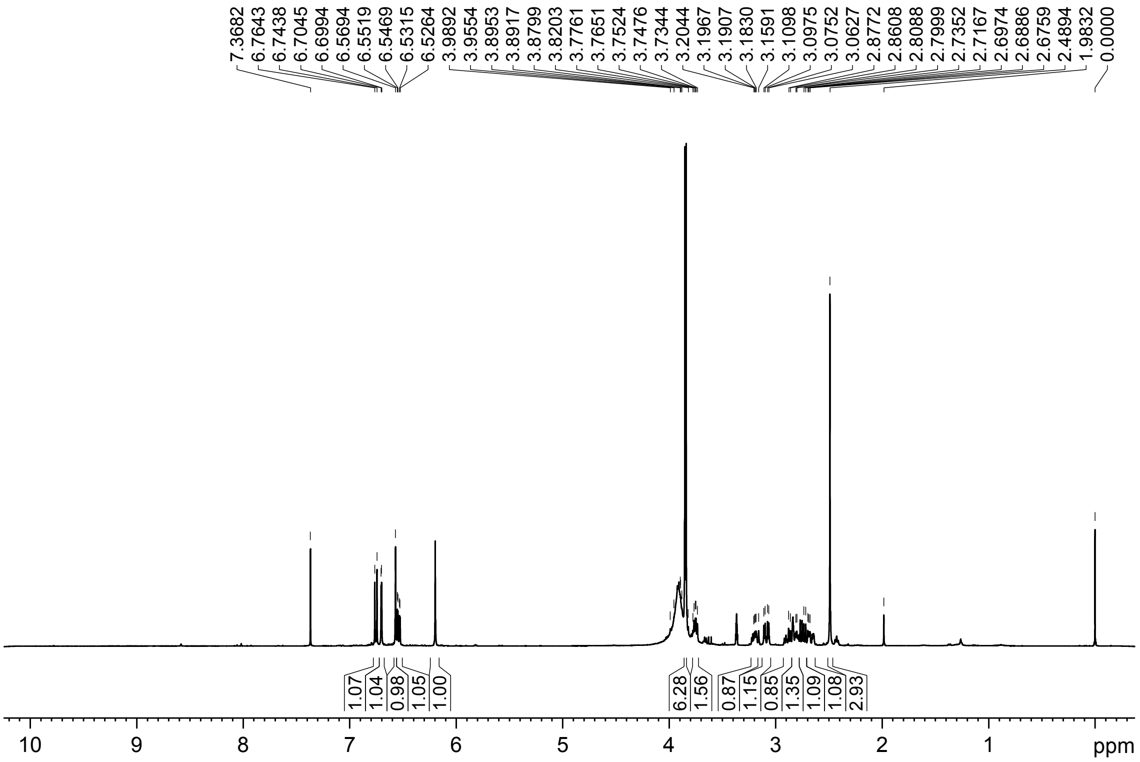

Figure S35. ^{1}H NMR spectrum of alkaloid **(+)-reticuline** ($CDCl_3$ + drops of CD_3OD at 400 MHz).

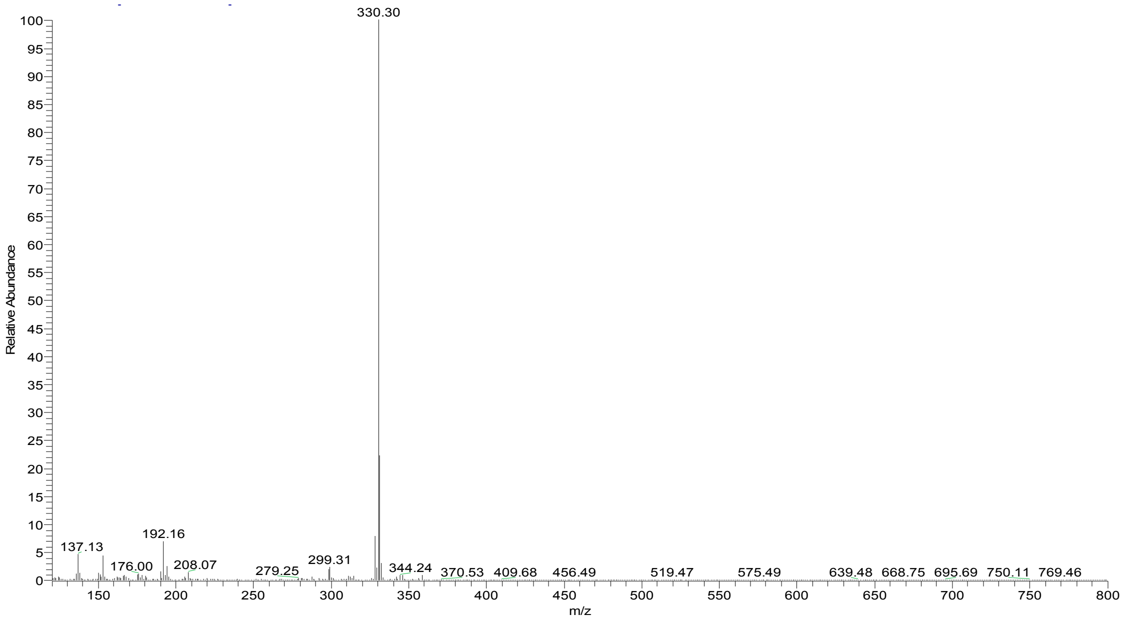

Figure S36. MS of alkaloid **(+)-reticuline** (*m*/*z* 330.30 $[M + H]^+$).

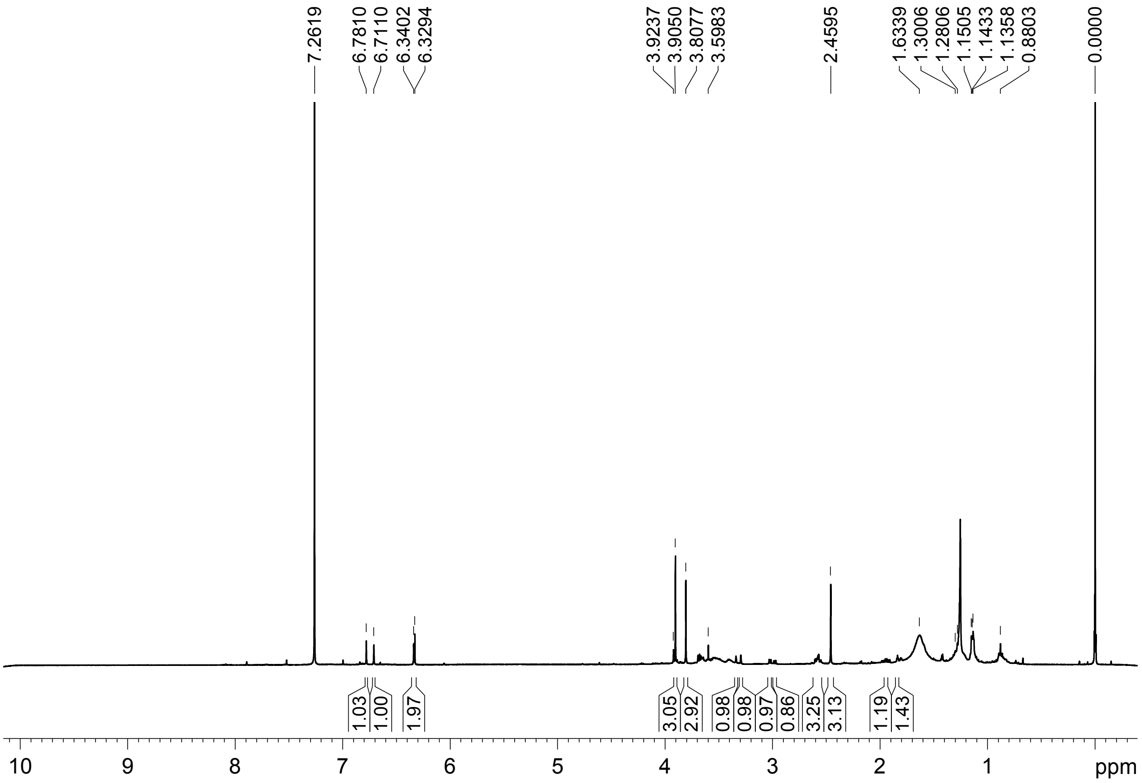

Figure S37. ^{1}H NMR spectrum of alkaloid **(+)-flavinantine** ($CDCl_3$ + drops of CD_3OD at 400 MHz).

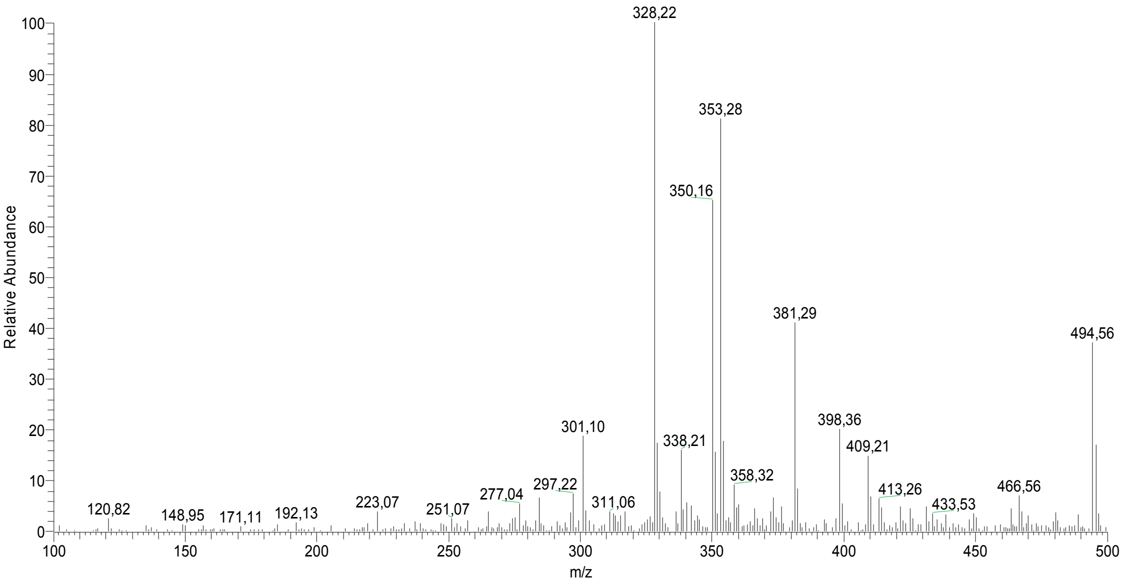

Figure S38. MS of alkaloid **(+)-flavinantine** (m/z 328.22 $[M + H]^+$).

Table S1: ^{1}H NMR data for alkaloids from the stem *X. laevigata* (δ in ppm and *J* values in (Hz))

no.	Roemerine [a]	Glaucine [a]	Xylopine [a]	Norglaucine [a]	Norpurpureine [a]	*N*-methyllaurotetanine [a]	Norpredicentrine [b]	Calycinine [c]
3	6.57 s	6.59 s	6.54 s	6.61 s		6.59 s	6.66 s	6.61 s
4	2.67 m	2.72 m	2.71 m	3.06 m	2.83 m	2.70 dd (16.3; 3.6)	3.16 m	2.70 ddd (16.0; 4.0; 1.1)
	3.14 m	3.26 m	3.03 m	2.73 m	2.83 m	3.15 m	2.86 m	3.07 ddd (16.0; 12.3; 5.8)
5	2.57 m	3.19 m	3.40 m	3.45 m	3.41 m	3.06 m	3.59 m	3.41 ddd (12.1; 5.8; 1.1)
	3.07 m	2.68 m	3.02 m	2.06 m	2.94 m	2.55 m	3.19 m	3.00 ddd (12.3; 12.1; 4.0)
6a	3.23 m	3.22 m	4.00 dd (14.1; 5.0)	3.90 m	3.83 m	3.07 d (13.6)	4.10 d (13.8)	3.85 dd (13.6; 4.1)
7	3.17 dd (13.7; 4.1)	2.73 m	2.81 t (14.1)	2.86 m	2.70 m	2.54 m	3.00 m	2.76 dddd (13.7; 13.6; 1.2; 0.9)
	2.69 t (13.7)	3.04 dd (13.7; 3.6)	2.95 dd (14.1; 5.0)	2.79 m	2.78 m	2.97 dd (13.6; 4.0)	2.85 m	2.89 dd (13.7; 4.1; 0.5)
8	7.25 m	6.78 s	6.81 dd (2.7; 0.7)	6.76 s	6.78 s	6.78 s	6.90 s	6.41 ddd (2.7; 1.2; 0.7)
9	7.25 m							
10	7.32 m		6.86 ddd (8.6; 2.7; 0.7)					6.50 dd (2.7; 0.9)
11	8.07 d (8.0)	8.08 s	8.01 d (8.6)	8.11 s	7.97 s	8.03 s	8.05 s	
OCH_3-1		3.65 s		3.66 s	3.73 s	3.64 s	3.62 s	
OCH_3-2		3.89 s		3.89 s	3.96 s	3.88 s		
OCH_3-3					3.91 s			
OCH_3-9		3.93 s	3.84 s	3.91 s	3.92 s		3.90 s	3.81 s
OCH_3-10		3.90 s		3.90 s	3.92 s	3.89 s	3.88 s	
N-CH_3	2.56 s	2.66 s				2.54 s		
(OCH_2O)1–2	6.10 d (1.4)		5.94 d. (1.4)					5.96 d. (1.4)
	5.95 d (1.4)		6.08 d. (1.4)					6.09 d. (1.4)

[a] The experiments were obtained at 303 K with TMS as internal reference (0.00 ppm) in $CDCl_3$ + drops of CD_3OD. [b] In CD_3OD + drops of $CDCl_3$. [c] In $CDCl_3$.

Table S2: ^{13}C NMR data for alkaloids from the stem *X. laevigata* (δ in ppm)

no.	Roemerine [a]	Glaucine [a]	Xylopine [a]	Norglaucine [a]	Norpurpureine [a]	*N*-methyllaurotetanine [a]	Norpredicentrine [b]	Calycinine [c]
1	142.9	144.9	142.2	144.6	149.5	144.3	144.8	138.9
1a	116.5	127.1	116.3	126.7	122.6	127.4	125.5	114.0
2	147.2	152.3	147.3	152.4	145.7	152.3	151.6	146.0
3	107.6	110.4	107.1	110.9	150.1	110.5	115.5	107.5
3a	126.5	125.5	125.7	128.5	122.8	128.7	127.9	127.6
3b	126.7	125.4	125.9	126.8	130.5	126.5	122.3	128.3
4	28.9	28.1	28.3	28.5	23.2	28.7	26.7	28.9
5	53.5	52.8	42.9	42.8	42.4	53.3	42.8	42.7
6a	62.2	62.2	53.3	53.6	53.9	62.6	54.3	53.5
7	34.5	33.7	36.5	36.3	36.1	33.8	34.9	38.2
7a	134.9	128.4	136.2	128.6	128.2	129.6	127.9	138.5
8	128.5	110.9	113.7	110.9	111.0	114.5	112.1	107.8
9	127.7	148.1	159.1	148.2	147.6	145.3	149.6	160.8
10	127.4	147.6	112.5	147.6	147.6	146.1	149.0	102.7
11	127.2	111.6	128.6	111.8	111.5	111.9	112.6	154.9
11a	131.1	124.2	133.8	124.5	124.5	123.7	125.2	110.9
OCH_3-1		60.2		60.3	60.7	60.2	60.3	
OCH_3-2		55.7		55.9	61.1	56.0		
OCH_3-3					60.5			
OCH_3-9		55.9	55.4	55.9	55.9		56.3	55.3
OCH_3-10		55.9		55.9	56.1	56.2	56.5	
N-CH_3	43.6	42.9				43.6		
(OCH_2O)1–2	101.0		100.8					100.2

[a] The experiments were obtained at 303 K with TMS as internal reference (0.00 ppm) in $CDCl_3$ + drops of CD_3OD. [b] In CD_3OD + drops of $CDCl_3$. [c] In $CDCl_3$.

Table S3. Specific rotation of alkaloids from *X. laevigata*.

**Compounds**	$[\alpha]_D^{20}$	*c*
Roemerine	−11.0°	0.2133
Anonaine	+12.9°	0.2700
Glaucine	+16.9°	0.0800
Xylopine	+42.9°	0.2133
Norglaucine	+181.7°	0.0666
Norpurpureine	+91.2°	0.0600
N-methyllaurotetanine	+28.3°	0.0733
Norpredicentrine	+81.4°	0.1333
Calycinine	+98.0°	0.1000
Laurotetanine	+54.6°	0.0333
Xylopinine	-207.9°	0.0266
Discretine	+45.2°	0.2066
Corytenchine	−3.7°	0.0533
Discretamine	+192.4°	0.0666
Reticuline	+74.1°	0.1733
Flavinantine	+45.5°	0.0530

Note: All the measured were realized in $CHCl_3$
